# Tumor Microenvironment Drives the Cross-Talk Between Co-Stimulatory and Inhibitory Molecules in Tumor-Infiltrating Lymphocytes: Implications for Optimizing Immunotherapy Outcomes

**DOI:** 10.3390/ijms252312848

**Published:** 2024-11-29

**Authors:** Ornella Franzese

**Affiliations:** Department of Systems Medicine, University of Rome “Tor Vergata”, Via Montpellier 1, 00133 Rome, Italy; franzese@uniroma2.it

**Keywords:** co-stimulatory molecules, inhibitory molecules, immune checkpoint, T cells, tumor micro-environment, cross-talk, T-cell activation, axis, immune checkpoint blockade

## Abstract

This review explores some of the complex mechanisms underlying antitumor T-cell response, with a specific focus on the balance and cross-talk between selected co-stimulatory and inhibitory pathways. The tumor microenvironment (TME) fosters both T-cell activation and exhaustion, a dual role influenced by the local presence of inhibitory immune checkpoints (ICs), which are exploited by cancer cells to evade immune surveillance. Recent advancements in IC blockade (ICB) therapies have transformed cancer treatment. However, only a fraction of patients respond favorably, highlighting the need for predictive biomarkers and combination therapies to overcome ICB resistance. A crucial aspect is represented by the complexity of the TME, which encompasses diverse cell types that either enhance or suppress immune responses. This review underscores the importance of identifying the most critical cross-talk between inhibitory and co-stimulatory molecules for developing approaches tailored to patient-specific molecular and immune profiles to maximize the therapeutic efficacy of IC inhibitors and enhance clinical outcomes.

## 1. Introduction

This review aims to explore some of the complex mechanisms that shape the T-cell mediated antitumor immune response, with a particular focus on the critical balance between co-stimulatory and inhibitory pathways. This balance is essential for maintaining self-tolerance and controlling immune response type, strength, and duration, as extensively documented in the literature [1,2,3,4]. Indeed, as appropriately reported by Fuertes Marraco et al., pathways mediated by inhibitory immune checkpoints (ICs) have co-evolved with co-stimulatory molecules as pivotal immune regulators to maintain immune homeostasis and prevent autoimmune reactions [2,5].

The achievement of an effective antitumor response relies on the activation of tumor-specific effector T cells, circulating between the blood and lymphoid tissues and ultimately infiltrating the immunosuppressive tumor microenvironment (TME) [6,7], where they may undergo sub-optimal priming and become predisposed to dysfunction due to various local intrinsic and extrinsic factors. Antigen-mediated stimulation within the TME fosters antitumor responses while simultaneously promoting T-cell exhaustion [8]. Consequently, dysfunctional T cells may emerge from an initially responsive subset, highlighting the necessity of being replaced by a less dysfunctional, long-lived population of tumor-reactive T cells. A key question is whether tumor-infiltrating T cells (TILs) reactivated by IC blockade are represented by resident lymphocytes or derive from T cells migrating from the peripheral blood, a process defined as a clonal replacement [9,10].

Furthermore, in the TME, malignant cells often express IC molecules that can engage their ligands on T cells, contributing to the dysregulation of antitumor immune response and tumor uncontrolled growth [11].

An optimal T-cell response requires the activation of the T-cell receptor (TCR) complemented by co-stimulation through stimulatory checkpoint pathways [12,13]. Conversely, inhibitory signals hinder this process by impairing T-cell activation and cytokine production. While inhibitory molecules serve as checkpoints to regulate immune responses and maintain tolerance, cancer cells can exploit these pathways to evade immune detection, promoting disease progression [14,15]. Thus, a delicate balance between stimulatory and inhibitory signals is essential for mounting an effective antitumor immune response while maintaining immune homeostasis and preventing autoimmune reactions [4,16].

The revolutionary impact of IC blockade (ICB) on the therapeutic landscape for advanced solid and hematologic cancers has led to a profound transformation in conventional tumor treatment approaches [17]. Nevertheless, despite the groundbreaking impact of the ICB approach, its efficacy is still limited, with only 20–30% of treated patients exhibiting a positive response. Thus, one of the most urgent challenges lies in identifying biomarkers predictive of clinical effectiveness and combined therapies able to overcome ICB resistance [18]. Stratifying patients based on specific molecular and immune features is crucial for identifying the most effective treatment, aligning with the current precision medicine paradigm, and maximizing clinical benefits.

ICBs enhance antitumor responses by reinvigorating tumor-reactive CD8^+^ T-cell clonotypes [19]. However, understanding the mechanisms involved and identifying the specific T-cell sub-populations that primarily undergo re-invigoration is of critical importance. Monoclonal antibodies (mAbs) targeting Programmed Cell death Protein-1 (PD-1) or its ligand PD-L1 can rescue T cells from exhaustion and restore their ability to mount an immune response against cancer cells. Due to their significant success in clinical trials, several anti-PD-1 mAbs, including nivolumab, pembrolizumab, and others, as well as anti-PD-L1 mAbs such as atezolizumab, durvalumab, and avelumab, have been approved for the treatment of different cancers [20].

However, the low response rate of anti-PD-1/PD-L1 therapies remains a challenge. One reason for this drawback is that, for most cancer patients, the PD-1/PD-L1 checkpoint is not the sole factor limiting antitumor immune responses [21], and blocking this pathway alone is often insufficient to regenerate an effective antitumor immune response [22]. A critical issue lies in the expression of several co-stimulatory and inhibitory molecules across different T-cell types within the TME [3,23], a complex milieu composed of cancer, stromal, endothelial, and innate immune cells such as myeloid cells, natural killer (NK), and innate lymphoid cells. Each cell type can differently contribute to the immunosuppressive or immunostimulatory features of the TME [24].

This complexity may necessitate a multifaceted approach to immunotherapy, targeting not only the PD-1/PD-L1 pathway but also other co-stimulatory and inhibitory signals within the TME. Mounting evidence suggests that ICIs amplify the antitumor immune responses by targeting not exclusively effector T cells but also other cell types within the TME [25]. Moreover, the versatile nature of some receptors, which can act as T-cell inhibitors or co-stimulators [26], often depending on the specific partner engaged, further complicates this landscape.

The initial model of CD28 and CTLA-4 functions, mediated by their interaction with B7-1 (CD80) or B7-2 (CD86) molecules, laid the groundwork for understanding the broader significance of co-stimulation and inhibition [27]. However, the canonical two-signal model of naive T-cell activation has evolved, acknowledging the multifaceted roles of secondary signals in modulating TCR signaling, differently affecting the distinct T-cell subsets.

Therefore, the dynamic relationship between the heterogeneous cell populations within the TME and the differential expression of co-stimulatory and inhibitory molecules creates a complex network that shapes the immune response to cancer. A comprehensive understanding of the diverse structure and function of these molecules and the outcomes arising from their individual and combined expressions and cross-talk is crucial for developing effective, patient-tailored immunotherapeutic strategies.

While acknowledging the value of a thorough discussion of ongoing clinical trials, a comprehensive review of these studies lies beyond the scope of this paper, as numerous excellent reviews have already addressed this topic. However, selected trials have been highlighted to contextualize the biological implications of immunological cross-talk. The primary focus of this review is on the mechanistic insights into the complex interplay between key co-stimulatory and inhibitory molecules and how this shapes T-cell behavior within the TME for therapeutic benefit.

## 2. The Co-Stimulatory Signals and Their Role in Immunotherapy: Successes and Challenges in Modulating Co-Stimulatory Signals for Therapeutic Benefit

In the absence of the co-stimulation required alongside TCR activation, T cells become anergic [28]. T-cell activation requires two to three critical signals. The first signal is initiated by antigen recognition through the TCR, while a second co-stimulatory signal generally involves the interaction between the co-stimulatory molecule CD28 expressed on T cells and B7 ligands present on antigen-presenting cells (APCs) [27,29]. T-cell cytokine receptors can provide a third signal, which further modulates the immune response [30]. Notably, memory and activated T cells may require lower levels of these signals to proliferate as compared with naïve T cells, proliferating and producing effector molecules at a higher extent compared with naive cells [31].

Essential proteins are phosphorylated upon TCR engagement, facilitated by non-receptor kinases such as tyrosine kinase LCK. This phosphorylation cascade involves ZAP-70 (Zeta-Chain-Associated Protein kinase 70), which phosphorylates LAT (Linker of Activated T cells), creating a platform for recruiting other factors in the activation pathway [32]. Recruitment of PLCγ-1 (phospholipase Cγ-1) is vital for phosphatidylinositol metabolism, while GADS (Grb2-related Adaptor Downstream of SHC) and Lymphocyte cytosolic protein 2 (SH2 domain-containing leukocyte protein of 76 kDa), also known as LCP2 or SLP-76, support cytoskeletal rearrangement. The MAPK cascade, initiated by GRB-2 (Growth factor receptor-bound protein 2)/SOS leads to ELK-1 (ETS Like-1 Protein Kinase) activation and subsequent FOS expression. Additionally, DAG (diacylglycerol) activates PKC and NF-κB which regulate gene expression. Proteins like VAV-1, NCK, and RHO further modulate T-cell cytoskeletal changes [29].

Co-stimulatory molecules are crucial for optimal T-cell activation and are finely tuned by inhibitory signals. Co-stimulation primarily involves interactions between CD28 family receptors and B7 family ligands. Over the last two decades, several critical molecules within these families have been identified. Notable members of the CD28 receptor family include CD28, CTLA-4 (Cytotoxic T-lymphocyte Associated Protein 4, CD152), PD-1 (CD279), ICOS (Inducible T-cell Costimulator, CD278), and BTLA (B- and T-lymphocyte Attenuator). Prominent ligands expressed in APCs belong to the B7 family, with CD80 and CD86 being central players [29]. Other molecules can transmit both positive and negative signals, modulating the T-cell response alongside the antigen-specific signal.

Co-stimulatory molecules of the Tumor Necrosis Factor receptor (TNFR) superfamily also play a crucial role in regulating immune responses. These molecules enhance T-cell activation, survival, and differentiation, which are all essential for mounting effective responses against tumors [33]. Members of the TNFR superfamily, such as CD27, CD40, OX40 (CD134), 4-1BB (CD137), and GITR (Glucocorticoid-Induced TNFR-related protein), interact with their respective ligands (CD70, CD40L, OX40L, 4-1BBL, and GITRL) to deliver positive signals that boost T-cell functions. These interactions promote T-cell proliferation and cytokine production, while contributing to the generation of memory T cells, crucial for providing long-term immunity. Furthermore, co-stimulatory signals from the TNFR superfamily can influence other immune cells, such as dendritic cells (DCs), B cells, and macrophages, orchestrating a coordinated and effective immune response [34].

A better understanding and manipulation of these co-stimulatory pathways can lead to more effective immunotherapeutic strategies, potentially improving outcomes for cancer patients.

### 2.1. CD28

**Figure 1:**
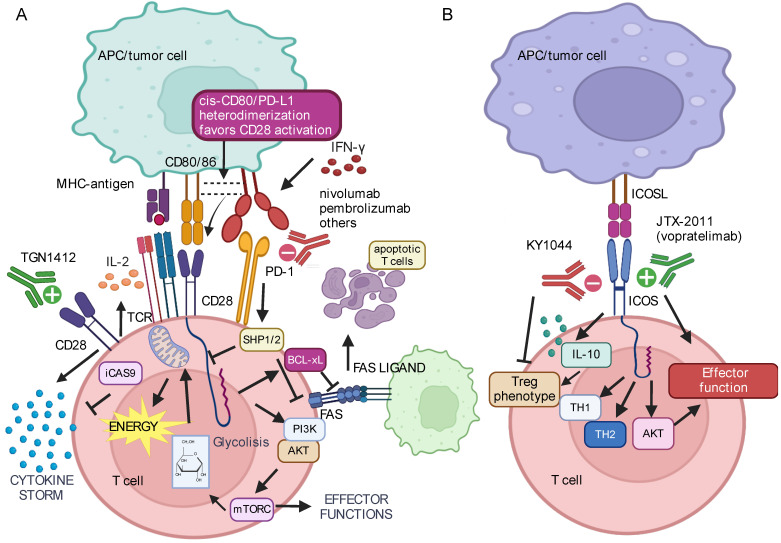
Highlighted here are key aspects of the complex roles and interactions of co-stimulatory molecules CD28 and ICOS in regulating T-cell activation within the TME, as discussed in the main text. Major ligands and intracellular signaling pathways are depicted, emphasizing their roles in modulating the antitumor immune response. Given the critical importance of this interaction, illustrated are also key features of the cross-talk between CD28 and the inhibitory molecule PD-1. (**A**) During T-cell activation, CD28 engages with B7 molecules CD80 and CD86 on APCs to sustain T-cell activation and protect T cells from apoptosis, promoting glucose and mitochondrial metabolism, proliferation, and effector function. PD-L1/CD80 cis-heterodimerization on APCs can preserve the CD80 capacity to activate CD28 while repressing the inhibitory activity of PD-1 and CTLA-4. CD28 is also a primary target downstream of PD-1. Monoclonal anti-CD28 superagonist TGN1412 activates T cells but induces a severe cytokine storm; (**B**) ICOS binds to its unique ligand ICOSL improving T-cell activation through activation of AKT kinase, but it also induces the generation, proliferation, and survival of Tregs. Agonist anti-ICOS mAb vopratelimab possesses significant antitumor efficacy. Antagonist mAb KY1044, aimed at depleting ICOS^high^ Tregs, also has shown encouraging clinical activity. The red circle with a minus sign represents inhibition of receptor activity and of its interaction with the ligand, the green circle with a plus sign indicates receptor activation. The pointed arrow indicates activation, the flat-tipped arrow indicates inhibition. Created in BioRender. Franzese, O. (2024) https://BioRender.com/l39g984, accessed 23 November 2024.

Initially described by Hansen in 1980 as a T-cell surface antigen, the CD28 co-stimulatory molecule is constitutively expressed in approximately 30–50% of human CD8^+^ T cells and nearly all CD4^+^ T cells. During T-cell activation, CD28 engages with B7 molecules CD80 and CD86 on APCs to sustain T-cell activation (Figure 1A). This process is followed by a substantial decrease of CD28 mRNA levels and surface expression, temporarily preventing T-cell restimulation, thus influencing the duration and intensity of immune responses [27].

Activation of CD28 triggers significant changes in T cells at epigenetic, transcriptional, and post-translational levels, exerting control over numerous T-cell functions [35]. Indeed, beyond its regulatory roles, CD28 is integral to various T-cell activities, including cellular reorganization, and cytokine and chemokine production, facilitating internal biochemical processes such as phosphorylation, transcription, and metabolism, all of which are vital for T-cell growth and specialization. Among the B7 partners of CD28, CD86 is consistently expressed on APCs, while CD80 is barely present in resting cells and is upregulated during inflammation. The interaction between CD28 and its ligands stimulates the production of significant levels of interleukin (IL)-2 (Figure 1A) and survival factors crucial for T-cell viability, proliferation, and differentiation [35].

The generation of a mature immune synapse initiates lymphocyte activation, concentrating the TCR receptors facilitated by the interaction of adhesion molecules with MHC complexes. CD28 signaling enhances the initial conjugation of T cells and APCs (including tumor cells), playing a critical role in cellular adhesion, which is crucial during the early activation stages [36].

Signaling from the TCR complex alone does not permanently activate T cells and can lead to anergy [28], or programmed cell death, especially under high antigen load and the absence of pro-inflammatory factors, such as anti-apoptotic cytokines IL-2, IL-7, and IL-15 [37]. CD28 activation also protects T cells from FAS-induced apoptosis by activating the AKT/PI3K pathway, inhibiting caspase (CAS)-8 recruitment, and upregulating BCL-xL expression to promote lymphocyte survival (Figure 1A). Moreover, while activation solely via the TCR does not initiate metabolic reprogramming, potentially leading to metabolic inactivity, T-cell co-stimulation via CD28 enhances the PI3K/AKT/mTORC1 pathway activation, promoting heightened glucose and mitochondrial metabolism, vigorous proliferation, and effector function (Figure 1A) [38].

CD28 also serves as a primary target downstream of the PD-1-mediated inhibitory signal (Figure 1A) [39,40] and characterizes a pre-exhausted CD8^+^ T-cell population prone to re-invigoration by ICB therapies. Additional studies have reported that CD28^+^CD8^+^ TILs exhibit higher responsiveness to PD-1 blockade, independent of CD28 and B7 interaction [41]. This observation may suggest a role for CD28 as a responsive biomarker to PD-1 blockade rather than being essential for their re-invigorating effects, highlighting the complexity of the immune response to cancer immunotherapy. Cytokines like IL-15 can re-invigorate PD-1 blockade in unresponsive CD28^−^ PD-1^+^CD8^+^ TILs [41]. Indeed, various IL-15 preparations are currently tested in clinical trials in combination with PD-1 blockade to treat Non-Small Cell Lung Cancer (NSCLC) [42].

CD28 expression has also been proposed as a prognostic factor for ICB response [41] and as a critical factor likely to deliver feasible biomarkers of ICB response [43,44]. While impacting the TCR signal, CD28 also controls T-cell tissue localization [45], promoting the egress from lymphoid tissue and relocation to inflammatory sites. This is noteworthy also because long-term TIL persistence correlates with the expression (or re-expression) of CD28 [46].

Notably, recent investigations have reported a more significant accumulation of PD1^+^CD28^+^ T cells at tumor sites in NSCLC patients [44], likely mediated by the impact of CD28 on T-cell tissue localization following antigen priming.

A correlation has been established between elevated levels of peripheral CD8^+^CD28^+^ T cells and prolonged progression-free survival (PFS) in metastatic breast cancer patients, whereas a high frequency of CD8^+^ T cells lacking CD28 is associated with shortened PFS [47].

The expression of CD28 has also been related to the assessment of the prognosis of lung cancer. Indeed, in NSCLC, a higher frequency of peripheral CD8^+^CD28^−^ T cells has been observed compared to healthy controls [47,48,49,50]. Similarly to what was reported for breast cancer patients, high peripheral CD8^+^CD28^+^ T-cell levels are linked to better overall survival (OS) and PFS in adenocarcinomas, whereas squamous cell carcinomas (SCCs) with a high frequency of CD8^+^CD28^−^ T cells tend to have worse OS and PFS [51].

However, in lung cancer prognosis assessment, features related to the TME must also be considered, regulating biological behaviors and outcomes. In particular, in young patients, CD28 has been associated with poor disease-free survival (DFS) but longer OS [52]. Higher CD28 expression in primary tumor tissues suggests inhibition of T-lymphocyte activity and correlates with an earlier TNM stage and fewer metastatic lymph nodes [52]. With tumor progression, the loss of CD28 expression in metastatic tissue indicates its involvement in metastatic mechanisms. The poor DFS observed in young LUAD patients associated with higher CD28 or PD-L1^+^CD28^+^ T-cell levels has prompted Authors to suggest that high basal CD28 levels may exhaust the ability to reverse tumor immune suppression, likely due to higher levels of chronic activation, resulting in immune escape and poor DFS. Conversely, increased CD28 expression correlates with longer OS, indicating different effects on lung cancer prognosis [52].

Monoclonal anti-CD28 superagonists, such as TGN1412, can activate T cells by binding to the C”D loop of the CD28 receptor, unlike other CD28-targeting Abs that bind near the natural ligand binding site. TGN1412, which expands T cells ex vivo without additional TCR stimulation, underwent extensive preclinical testing. Preclinical studies showed a well-tolerated T-cell expansion without measurable pro-inflammatory reactions, suggesting its potential for treating autoimmune diseases by activating regulatory T cells (Tregs). However, when tested in humans, TGN1412 caused severe cytokine storm (Figure 1A) and adverse reactions, leading to multiorgan failure and requiring intensive care for the volunteers within eight hours of infusion. Consequently, it was immediately withdrawn from Phase I clinical trials [53]. It has been suggested that the use of targeted delivery mechanisms, such as nanoparticle carriers or Ab-drug conjugates, to ensure that CD28 agonists are delivered specifically to the TME could minimize systemic exposure or incorporate safety switches in T cells, such as inducible CAS-9 (iCAS-9) (Figure 1A) [54], which can be activated to eliminate T cells in the presence of severe adverse effects, providing a fail-safe mechanism.

In summary, while CD28 agonism holds therapeutic potential, its application requires significant modifications to avoid the severe adverse effects observed in the TGN1412 trial. By improving selectivity, targeting, and safety mechanisms, the therapeutic benefits of CD28 agonism might be harnessed more safely and effectively.

### 2.2. ICOS

As a member of the CD28 superfamily, ICOS shares several structural and functional features with CD28, although with significant distinctions. At variance with CD28, ICOS is not consistently expressed in resting T cells, suggesting its unique role in regulating adaptive T-cell responses. ICOS is recognized for its unique ligand, ICOSL (Figure 1B), mainly expressed on APCs and non-lymphoid tissue cells, and its inability to bind with B7 molecules CD80/CD86, providing an interesting study objective [55].

Notably, the overexpression of ICOS can influence both T helper (Th)1 and Th2 immune responses, as well as Tregs, playing a critical role in maintaining immune balance during inflammation. Indeed, recent reports have shed light on the dual role of ICOS signals in immune responses, implicating ICOS^+^ T cells in both pathological effects and anti-inflammatory responses, particularly through ICOS^+^ Tregs. Indeed, the ICOS signaling pathway enhances the generation, proliferation, and survival of Tregs, with evidence suggesting that ICOS^+^ Tregs possess superior suppressive abilities, partly due to the induction of IL-10 (Figure 1B) [56]. This complexity makes it challenging to isolate the impact of ICOS on immune responses, given its essential role also in supporting optimal effector memory T-cell generation and function [55,57]. However, the mechanisms underlying these events remain incompletely defined and require further investigation.

Considering the, at least partially, overlapping functions of CD28 and ICOS-mediated signals, it is plausible that the interplay between these co-stimulatory signals, along with contributions from other cell surface proteins like the TCR and cytokine receptors, determines the significant impact of ICOSL signals in shaping T-cell function and differentiation. Different signals impact the quality of APCs by modulating their CD80, CD86, and ICOSL expression, thus influencing the balance between the ICOS and CD28-mediated co-stimulation received by T cells. In CD8^+^ T cells, ICOS has been recognized to enhance activation, particularly when CD28 expression is reduced or absent [58]. More recent research has linked ICOS to the functional advantage of CD8^+^ antigen-specific T-cell clones isolated from melanoma patients through activation of AKT in the absence of CD28 (Figure 1B) [59], suggesting a potential role in cancer immunotherapy. However, challenges remain, especially concerning the diverse contexts in which T cells operate. The functional advantage provided to peripheral CD28^−^ T cells by ICOS has been confirmed in the peripheral blood but not at the tumor site of NSCLC cancer patients [44]. Considering that a subgroup of CD8^+^CD28^−^ T cells has been proposed as a distinctive Treg subpopulation [60,61], it has been suggested that it cannot be ruled out that a fraction of intra-tumor CD8^+^PD1^+^CD28^−^ T cells may have switched to a Treg-like phenotype, likely driven by the local immunosuppressive milieu [44]. Therefore, the clinical outcome of ICOS agonist therapy in cancer immunotherapy may face challenges due to the dual nature of the ICOS/ICOSL axis as either enhancer of antitumor T-cell reactions or inducer of pro-tumor responses, depending on the critical expression and functional role of ICOS on Tregs and other T-cell subtypes. This evidence suggests a strong need for an in-depth analysis of the ICOS^+^ T-cell context to successfully target the ICOS/ICOSL axis as a potential tool for cancer immunotherapy.

Different mAbs targeting this pathway have been explored for cancer immunotherapy [62]. Many studies are reported by Solinas et al. [63]. However, as above stated, in cancer immunotherapy, heightened Treg activity potentially promoted by ICOS agonist therapy could sustain Treg-mediated immune suppression, inhibiting effector T-cell responses, thereby limiting the therapeutic benefit.

Therefore, it has been suggested to employ strategies aimed at mitigating the negative impact of ICOS agonist therapy on Tregs, likely including combination therapies with agents targeting Tregs directly (e.g., anti-CTLA-4 or anti-PD-1/PD-L1) or refining ICOS agonist dosing to minimize Treg activation while maximizing effector T-cell responses [63]. Indeed, ICOS exhibits less potency when targeted alone compared to other pathways commonly addressed in cancer immunotherapies. At the same time, concurrent activation of CTLA-4 and ICOS achieves a more robust antitumor response compared to treatment with anti-CTLA-4 alone. Treatment with either anti-CTLA-4 or anti-PD-1 mAbs in both mice and patients results in an expansion of ICOS^high^ (FOXP3^–^) CD4^+^ and CD8^+^ T-cell subsets, indicating an overall favorable treatment effect and proposing ICOS^high^ T cells as a critical biomarker for assessing clinical response [63].

Anti-ICOS Abs, including the humanized JTX-2011 (vopratelimab), have demonstrated significant antitumor efficacy (Figure 1B) and durable protection in preclinical syngeneic mouse tumor models. Improved therapeutic outcomes have been observed when combining ICOS Abs with anti-PD-1 and anti-CTLA-4 therapies. Building on these preclinical findings, vopratelimab has now advanced through clinical development for the treatment of solid tumors [64].

In the trial INDUCE-1 (NCT02723955), the use of the ICOS agonist Ab feladilimab administered either alone or in combination with PD-1 blockade in patients with advanced solid tumors has yielded encouraging outcomes in terms of tolerability, toxicity profile, and clinical efficacy [65]. The ICONIC trial (NCT02904226), which investigated vopratelimab alone or in combination with nivolumab in patients with relapsed/refractory tumors has reported safety, tolerability, and potential antitumor effects, particularly in heavily treated gastric cancer and triple-negative breast cancer [66]. However, while ICOS agonist therapy holds promise in enhancing antitumor immunity, its potential negative impact due to the critical expression and functional role of ICOS on Tregs underscores the complexity of immune regulation in cancer.

On the other hand, KY1044, a fully human IgG1 anti-ICOS Ab (Figure 1B), aimed at depleting ICOS^high^ Tregs and enhancing effector T cells in the TME, was administered in a Phase 1/2 trial (NCT03829501) to patients with advanced/metastatic malignancies, who received escalating doses of KY1044 alone and in combination with atezolizumab. The treatment was well tolerated without dose-limiting toxicities. Preliminary data showed encouraging clinical activity, including objective responses in triple-negative breast cancer and other malignancies [67]. The Phase I expansion and Phase II part of the study is currently ongoing (NCT03829501, https://clinicaltrials.gov/, last accessed 31 October 2024). Notably, in a melanoma lung metastasis model, ICOS knockout in Tregs has been reported to reduce tumor burden and increase Ly108^+^ Eomes^+^ CD8^+^ T cells, suggesting that ICOS-expressing Tregs inhibit cytotoxic T-cell maturation [68].

Addressing the challenges suggested by these observations is crucial and urgent for optimizing the clinical utility of targeting ICOS in cancer immunotherapy. Indeed, biomarker-driven approaches may help to identify patients likely to benefit from ICOS agonist or antagonistic therapies and suggest that ICOS agonists may be more effective in tumors with low Treg presence or when combined with treatments that deplete tumor-infiltrating Tregs [68].

### 2.3. CD137/4-1BB

CD137, a member of the TNFSR family, possesses potent co-stimulatory capabilities, providing crucial pro-survival signals to activated T cells [69], while playing a critical role in cytokine release and promotion of T-cell effector functions [70].

**Figure 2:**
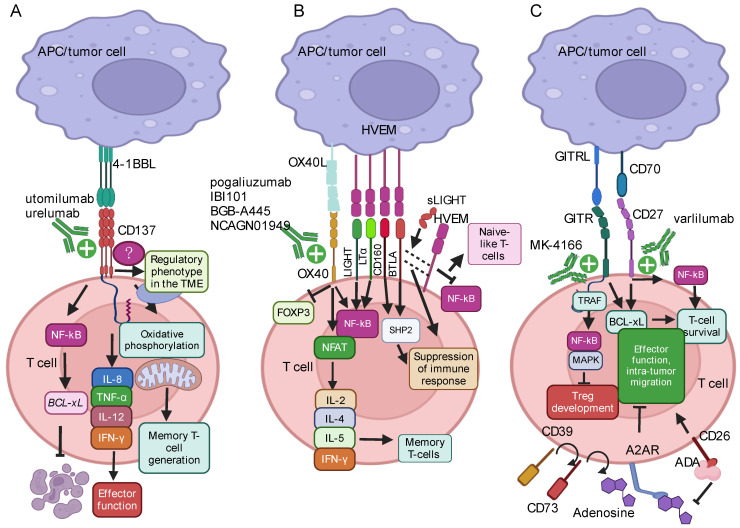
Key aspects of the complex roles and interactions of selected co-stimulatory molecules. Regulation of T-cell activation by CD137, OX40, the HVEM-BTLA-LIGHT axis, GITR, CD27, and the ADA/CD26 axis within the TME is illustrated, as described in the main text. Main ligands and intracellular signals are depicted, highlighting their roles in controlling antitumor immune response. (**A**) Upon engagement with 4-1BBL (TNFSF9), CD137 activates the NF-κB pathway, which upregulates BCL-xL, stimulates the production of Th1 cytokines IL-8, TNF-α, IL-12, IFN-γ and enhances oxidative phosphorylation and mitochondrial biogenesis, promoting long-term immune memory. Agonist mAbs targeting CD137 utomilumab and urelumab have potential antitumor efficacy; (**B**) Agonist anti-OX40 mAbs, including pogalizumab, BGB-A445, IBI101, and INCAGN01949, among others, are currently under investigation. HVEM interacts with multiple ligands (i.e., LIGHT, LTα, CD160, and BTLA), exerting distinct immune effects depending on the ligand. Engagement with LIGHT or LTα stimulates the NF-κB pathway. In contrast, interaction of HVEM with BTLA or CD160 suppresses immune responses through the recruitment of SHP-1/2 phosphatases. The cis interaction between BTLA and HVEM activates NF-κB, contributing to maintaining T cells in a naïve-like state, while a soluble form of LIGHT (sLIGHT) stabilizes the BTLA/HVEM in cis interaction, reinforcing this regulatory mechanism; (**C**) GITR supports T-cell survival and activation by increasing BCL-xL, NF-κB, and MAPK while inhibiting Treg-mediated suppressive activity. Anti-GITR agonistic Abs like MK-4166 impair Tregs and enhance effector response. CD27 contributes to T-cell activation and reduces FAS-L-mediated T-cell apoptosis by inducing BCL-xL. Agonist anti-CD27 mAb varlilumab improves T-cell activation. CD39 and CD73 collaborate in the generation of adenosine, contributing to the establishment of an immunosuppressive TME, while CD26 enhances T-cell activity, also by interacting with extracellular ADA. Extracellular adenosine impairs T-cell functionality by engaging with A2AR. The red circle with a minus sign represents inhibition of receptor activity and of its interaction with the ligand, the green circle with a plus sign indicates receptor activation. The pointed arrow indicates activation, the flat-tipped arrow indicates inhibition. Created in BioRender. Franzese, O. (2024) https://BioRender.com/r92x123 (accessed 22 November 2024).

Mascarelli et al., among others, have recently highlighted the role of CD137 in improving antitumor responses [71]. Upon engagement with its ligand, 4-1BBL (TNFSF9) (Figure 2A), expressed on activated APCs, B cells, macrophages, and other myeloid-derived cells, CD137 initiates signaling pathways within T cells. The cytoplasmic domains of CD137 bind to TNFR-associated factors [72], activating, among others, the NF-κB pathway, which upregulates survival genes like *BCL-xL* (Figure 2A) [73]. Furthermore, the CD137/4-1BBL axis stimulates the production of Th1 cytokines, such as IL-8, TNF-α, IL-12, and Interferon (IFN)-γ (Figure 2A), while suppressing Th2 molecules, resulting in increased T-cell activation, cytotoxicity, and DC maturation [71].

CD137 also serves as a surrogate marker of naturally occurring tumor-reactive T-cell specificity in tumors, facilitating the identification and isolation of live, human antigen-specific CD8^+^ T cells without tetramer staining [74].

Similarly to ICOS, CD137 enhances the functionality of peripheral CD8^+^PD-1^+^CD28^−^ T cells, providing a critical advantage during viral infections and in NSCLC patients [44,75]. However, this advantage can be lost upon transition to the NSCLC tumor site, akin to PD1^+^CD28^−^ T cells expressing ICOS [44]. Circulating CD137^+^ T cells have been shown to correlate with improved response to anti-PD1 immunotherapy in cancer patients [76]. At variance, intra-tumoral CD137 expression has been also identified as a conserved regulatory T-cell signature (Figure 2A) [77]. Again, these observations underscore how outcomes from different co-stimulatory molecules are influenced by their interplay with different molecules and cues specific to distinctive microenvironments.

Of relevance, CD28 and CD137 are among the most extensively studied co-stimulatory domains in CAR T-cell research. Clinically approved CAR T-cell therapies currently utilize one of these co-stimulatory domains. Although CAR T cells with either CD28 or CD137 co-stimulatory domains show similar antitumor efficacy in B-cell malignancies [78], preclinical models highlight significant biological differences. The 4-1BB signaling pathway enhances oxidative phosphorylation and mitochondrial biogenesis, promoting long-term immune memory (Figure 2A) [78]. At variance, the CD28 domain boosts T-cell activation, cytokine production, and proliferation, primarily enhancing immediate cytotoxicity and short-term cytokine secretion with increased glycolytic metabolism [78,79].

Unlike CD28, the 4-1BB domain is linked to sustained CAR T-cell responses by reducing exhaustion from persistent CAR signaling. As reported, CD28-CAR T cells exhibit antigen-independent CD3ζ phosphorylation and greater antigen-dependent activation at low antigen levels, leading to exhaustion [79]. Differently, CAR T cells with the 4-1BB domain tend to show better persistence, long-term functionality, and memory formation, which are beneficial in terms of long-lasting antitumor effects and reduction of tumor relapse [80]. Third-generation CAR T cells often combine both 4-1BB and CD28 domains to leverage the cytotoxicity and proliferation provided by CD28 with the enhanced memory and persistence of 4-1BB [81].

The use of mAbs targeting CD137 is extensively explored in cancer immunotherapy. Based on preclinical findings showing the therapeutic efficacy of a combinatorial approach including PD-1 blockade and CD137 activation in murine cancer models [82], a Phase I clinical trial (NCT02652455) evaluated the safety and feasibility of combining nivolumab with TIL adoptive cell therapy (ACT) cultured in the presence of CD137 agonism to improve culture success rates, growth kinetics, and CD8^+^ T-cell composition. The primary endpoint was to establish the recommended Phase II schedule for this combination treatment. It was hypothesized that administering nivolumab before surgical tumor harvest and during TIL generation would enhance T-cell trafficking to the tumor, improve TIL quality and persistence, and prevent tumor progression during the interval between harvesting and infusion [83]. Combination therapy was reported as safe and feasible for patients with metastatic melanoma while delivering critical insights for potential ACT improvements.

Among the primary agonists utilized in clinical settings, the IgG2 mAb utomilumab (PF-05082566) (Figure 2A) was investigated in a Phase Ib study (NCT02179918) conducted in advanced solid tumors in association with pembrolizumab. No dose-limiting toxicities were observed among 23 patients, with mostly mild to moderate adverse events and no treatment discontinuations. Pharmacokinetics and immunogenicity profiles were comparable whether drugs were administered alone or in combination [84], supporting further investigation.

Urelumab (BMS-663513) has demonstrated notable antitumor efficacy, although posing a risk of liver damage, potentially mediated by FcγRIIB-associated CD8^+^ T-cell activation. The administration of lower doses of urelumab, in combination with agents such as rituximab, cetuximab, and nivolumab, has shown promise in enhancing the host immune response while minimizing liver toxicity, particularly in the treatment of relapsing or refractory non-Hodgkin lymphomas [85,86].

However, two recent studies evaluating urelumab combined with cetuximab or nivolumab in patients with advanced solid tumors, including metastatic colorectal cancer, metastatic squamous cell head and neck carcinoma (SCCHN), melanoma, and NSCLC (NCT02110082; NCT02253992), have shown that the addition of urelumab at the described doses is tolerable, although the preliminary response rates did not demonstrate a clear additive benefit [86]. Despite this drawback, the positive pharmacodynamic effects observed with urelumab suggest that further investigation into other anti-CD137 agonist agents for cancer treatment is warranted.

### 2.4. OX40

OX40 (CD134) is a critical player in improving antitumor responses, as widely described [71]. OX40 expression has been identified across various immune cell subsets, including activated CD4^+^ and CD8^+^ T cells, Tregs, follicular helper T cells, NK cells, and neutrophils [71]. Upon TCR activation, OX40 expression on T cells is upregulated, a process that may be amplified by the CD28/CD80 engagement [87], and interacts with OX40 ligand (OX40L), primarily expressed on APCs (Figure 2B) [88], promoting transcriptional changes that modulate T-cell proliferation and survival.

OX40 possesses a cytoplasmic tail that engages with molecules involved in TNFR-associated signal transduction pathways and the activation of the NF-κB pathway [89], which indirectly increases the expression of apoptosis-suppressing proteins and prolongs cell survival. Additionally, it activates the T-cell nuclear factor (NFAT), resulting in increased production of cytokines such as IL-2, IL-4, IL-5, and IFN-γ (Figure 2B) [90].

While some data suggest the importance of OX40/OX40L signaling for primary and memory Th2 responses [91], there is good evidence indicating that OX40 co-stimulation plays a significant role in the Th1 response, inducing potent co-stimulatory signaling in both CD4^+^ and CD8^+^ T cells, with a more pronounced effect on the CD4^+^ compartment [92]. Nevertheless, OX40 co-stimulatory signaling can re-invigorate exhausted CD8^+^ T cells, restoring their proliferative potential and cytokine production while favoring IFN-γ expression in tumor-infiltrating immune cells, associated with favorable patient prognosis in small-cell lung cancer (SCLC) [71].

Additionally, agonistic OX40 signaling induces a BATF-3-dependent downregulation of FOXP3 expression in Tregs (Figure 2B) [93]. Since Tregs can dampen antitumor responses by inhibiting T-cell proliferation, OX40 co-stimulation may counteract the immunosuppressive activity of Tregs and prevent the conversion of effector CD4^+^ T cells into regulatory a phenotype [93].

In mice, treatment with OX40 agonist mAbs has been linked to the accumulation and increased survival of CD4^+^ T cells, enhanced priming of CD8^+^ T cells, and the promotion of memory T-cell development (Figure 2B). This treatment also disrupts immune tolerance, as demonstrated by tumor regression in preclinical models of sarcoma, melanoma, colon carcinoma, and glioma [71].

Clinical trials employing OX40 mAbs, such as 9B12 and MOXR 0916 (pogalizumab), have demonstrated an increased proliferation of CD4^+^ and CD8^+^ T cells, enhanced IFN-γ production, and amplified antitumor reactivity of T and B cells. Numerous clinical trials are exploring the application of co-stimulatory anti-OX40 mAbs as adjuvants in combination with chemotherapy, radiotherapy, and PD-L1 blockade in patients with advanced solid neoplasms, with OX40-expressing TILs correlating with better outcomes in NSCLC patients. Also, the development of new anti-OX40 Abs, such as IBI101, shows promising results in preclinical models with anti-PD-L1 therapy [71].

Combining agonist anti-OX40 Abs with radiotherapy or other ICIs like anti-CTLA-4 or PD-1 Abs holds promise in various cancers, including colon, pancreatic, and bladder cancers. In a recent study, OX40 agonist therapy has been reported to enhance T-cell proliferation and cytokine secretion in anti-PD-1 responders, with a clear increase in OX40 expression on T cells [94]. This combination therapy shows potential in enhancing the immune response to PD-1 blockade, thereby improving the outcomes for patients with NSCLC.

Advanced pancreatic ductal adenocarcinoma (PDAC) resists most therapies, including ICIs. The combination of anti-PD-1 and OX40 agonist mAbs has been reported to significantly increase survival in mice with pancreatic tumors, with nearly 100% surviving 225 days and becoming tumor-free [95]. This combination reduces Tregs and exhausted T cells while increasing memory T cells. Notably, high OX40 and low PD-1 levels in human PDAC correlate with better patient survival, suggesting that a dual approach targeting PD-1 and OX40 could enhance immune-based treatments for PDAC.

BGB-A445 is a novel anti-OX40 agonist mAb, which distinctively does not interfere with the binding of the natural OX40 ligand. Preclinical studies have shown that BGB-A445 exhibits antitumor activity both as an individual treatment and when combined with an anti-PD-1 mAb. Data from an ongoing Phase I of a multicenter, dose-escalation/expansion study (NCT04215978) evaluating BGB-A445, either as monotherapy or in combination with tislelizumab, in patients with advanced solid tumors like NSCLC, Head and Neck Squamous Cell Carcinoma (HNSCC), Nasopharyngeal Carcinoma (NPC), have been described by Desai et al. [96]. In the dose-escalation Phase of this study, BGB-A445, either alone or in combination with tislelizumab, was generally well tolerated at all the administered doses, with preliminary findings also indicating good antitumor activity. The study is now continuing into the dose-expansion Phase, focusing on patients with NSCLC and HNSCC. The estimated study completion date is November 2024 (https://clinicaltrials.gov/, last accessed 31 October 2024).

INCAGN01949, a fully human IgG1 anti-OX40 agonist mAb, was developed to boost tumor-specific immunity by activating effector T cells and depleting Tregs through Fcγ receptor engagement. A first-in-human Phase I/II open-label, non-randomized trial (NCT02923349) was aimed to assess the safety, tolerability, and preliminary efficacy of INCAGN01949 in patients with advanced or metastatic solid tumors. In the study, one patient with metastatic gallbladder cancer showed a partial response lasting 6.3 months, and 23 patients achieved stable disease, with one case exceeding 6 months [97]. However, INCAGN01949 did not significantly improve T-cell proliferation or activation or reduce circulating Tregs. Tumor biopsies also showed no consistent changes in effector T-cell infiltration or Treg reduction. In summary, INCAGN01949 monotherapy was safe for patients with advanced solid tumors, but its impact on tumor response and T-cell activity was limited. Also, the Phase I study ENGAGE-1, which evaluated the humanized IgG1 OX40 agonist mAbs GSK3174998 alone or in combination with pembrolizumab in patients with advanced solid tumors (NCT02528357), has indicated good toleration yet limited clinical activity, not supporting further development [98].

Further research is needed to evaluate the potential of combining INCAGN01949 and GSK3174998 with other therapies to enhance the therapeutic effects of OX40 agonism.

### 2.5. The HVEM/BTLA/LIGHT Axis

The BTLA/HVEM/LIGHT axis is a complex system involving the type I transmembrane protein HVEM from the TNFR family [99]. This receptor exhibits affinity towards ligands from diverse families, including other TNFSF members like LIGHT and lymphotoxin alpha (LTα), CD160, and BTLA (Figure 2B), thus potentially fine-tuning infectious, antitumor, and inflammatory autoimmune responses [99,100,101,102,103]. The HVEM engagement with either LIGHT or LTα stimulates positive immune responses via activation of the NF-κB pathway (Figure 2B) [101], providing the second signal for the co-stimulation of T and B cells, NK, and DCs, among others. Additionally, HVEM can stimulate B cells to produce immunoglobulins (Igs) and induce monocytes and neutrophils to produce cytokines, nitric oxide, and reactive oxygen species, also in the absence of primary signals [101].

Conversely, the interaction of HVEM with BTLA or CD160 suppresses immune responses [102,104] by recruiting protein tyrosine phosphatases SHP 1/2 (Figure 2B) [102]. Indeed, studies on HVEM/BTLA deficient mice have reported an increased T-cell responsiveness and heightened susceptibility to autoimmune disorders [105], implying that HVEM-mediated signaling primarily promotes immune suppression rather than immune stimulation [103,105,106,107]. In particular, the BTLA-HVEM complex has gained significant scientific interest as a crucial regulator in cancer immune contexts, as recently reviewed [101].

Notably, in the context of T cells, these molecules can interact either in cis (on the same cell) or trans (when expressed on different cells). Trans interaction between BTLA on T cells and HVEM on APCs impairs T-cell activation by inhibiting the phosphorylation of molecules involved in TCR signaling, thus suppressing T-cell proliferation and cytokine production. Of note, the BTLA/HVEM cis interaction, primarily observed in naïve T cells, inhibits the NF-κB pathway, maintaining T cells into a naïve-like state (Figure 2B). Interestingly, in vitro studies have revealed an additional function of the HVEM ligand, LIGHT, as a regulator of BTLA signaling. Indeed, the soluble form of LIGHT (sLIGHT) stabilizes the cis interaction of the BTLA/HVEM complex (Figure 2B). In contrast, membrane-bound LIGHT disrupts the cis BTLA-HVEM complex formation [105,108]. Thus, the BTLA/HVEM signaling pathway suitably exemplifies the critical importance of fine-tuned signals in modulating immune response intensity and duration.

### 2.6. GITR

GITR, a key player modulating T-cell responses and tumor immunity, also known as TNFRSF18, AITR, and CD357, is a member of the TNF superfamily [109]. In humans, GITR is found at high levels on FOXP3^+^ Tregs [110,111] and at a lower extent in different immune cells, including CD56^+^ NK cells, B cells, naïve, and memory T cells. GITR expression increases on Tregs and effector T cells upon activation. The natural ligand for GITR (GITRL, also known as TNFSF18) is mainly expressed on activated APCs and has been observed in specific tumor types. However, GITR engagement can support T-cell survival, at least in part, by increasing the expression of BCL-xL (Figure 2C), thus counteracting T-cell apoptosis [112]. The effects of GITR upon Tregs are more complex than those observed in conventional T cells, with in vitro studies reporting that GITR stimulation can reduce Treg-mediated immunosuppression by either protecting conventional effector T cells from Treg-mediated inhibition and directly reducing Treg suppressive activity [113,114,115,116].

From a signaling perspective, GITR, like other TNFRSF members, binds to TNFR-associated factor (TRAF) [117], activating intracellular pathways such as NF-κB and MAPK (Figure 2C) [118]. These pathways lead to the inhibition of the Treg-mediated suppressive activity and upregulation of metabolic pathways inducing cytokines critical for T-cell activation and proliferation [119,120]. Conversely, GITRL reverse signaling in APCs and tumor cells can have tolerogenic effects. However, due to its positive effects on T-cell responses, GITR has become a promising immunotherapeutic target [121], like other TNFRSF members.

Preclinical studies have shown a solid antitumor activity of GITR agonists in various tumor models, alone or in combination with other immunotherapies. The predominant mechanism underlying Treg loss has been reported to be cellular depletion [113], with minimal evidence of Treg conversion to a non-FOXP3-expressing population.

Further analysis of residual Tregs following anti-GITR mAb treatment has revealed preferential targeting of a highly activated subset of intra-tumoral Tregs characterized by CD44^high^ICOS^high^ expression, leading to their preferential elimination and a concurrent reduced suppressive phenotype displayed by spare Tregs [113,122]. These changes in the Treg population are accompanied by a shift in the composition of intra-tumoral CD8^+^ T cells towards a more functional state, characterized by reduced expression of exhaustion markers such as PD-1 and LAG-3, depending on both agonist GITR signaling and Treg depletion.

Evaluation of the anti-human agonist GITR antibody MK-4166 (Figure 2C) in a humanized mouse cancer model has mirrored many effects observed in syngeneic mouse tumor models, including a reduction in Treg numbers and suppressive phenotype, alongside enhanced effector responsiveness [123]. However, limited clinical efficacy has been reported in cancer patients, possibly due to the context in which these agents have been evaluated or to the biological differences between human and mouse systems. Nevertheless, combined approaches with PD-1 blockade have shown some clinical responses, suggesting potential synergistic effects. Despite current limitations, the ongoing clinical development of GITR agonists indicates a persistent interest in exploring their therapeutic potential in cancer treatment. Several GITR monospecific agonist mAbs, including AMG-228, ASP1951, and TRX518, have been released [116], although strong immune heterogeneity has been reported by transcriptomic analysis of GITR and GITR ligands, with critical implications for GITR targeting approaches [124]. GITR-targeting compounds vary in terms of structure and function, with many tested in patients with advanced solid tumors, either as monotherapy or combined with PD-1 inhibitors or chemotherapy. Most have shown no unusual safety concerns, but only TRX518 has demonstrated single-agent activity. TRX518 is distinct because it stimulates GITR and blocks specific interactions, potentially preventing negative signaling.

A Phase I study (NCT04465487) has assessed the safety, tolerability, pharmacokinetics, pharmacodynamics, and antitumor activity of REGN6569, administered in combination with cemiplimab, in patients with advanced solid tumors lacking approved ICI therapies [125]. High-grade adverse events occurred in a good fraction of patients, and only a few patients had partial tumor responses. The trial has moved to a dose-expansion Phase, focusing on HNCC patients resistant to anti-PD-1 therapy (https://clinicaltrials.gov/, last accessed 31 October 2024). Key findings suggest that the specific Ab structure may explain differences between preclinical and clinical results.

A combination of anti-PD-1 and agonist GITR Abs has been reported to induce synergistic enhancement of CD8^+^ T-cell function and a robust survival benefit [126], along with reduced T-cell dysfunction and increased proliferation of T cells with a precursor effector memory (EM) phenotype, which can optimize cancer immunotherapy outcomes. Pharmacodynamic studies have confirmed significant effects on Tregs and other immune cells, confirming a signaling role for GITR in modulating Tregs in humans.

### 2.7. CD27

CD27, a member of the TNFR family (TNFRSF7), is consistently present as a disulfide-linked homodimer on CD4^+^ and CD8^+^ T cells, as well as a specific subset of NK cells. However, its expression increases upon T-cell activation. CD27 is activated through interaction with CD70 (TNFSF7, also known as CD27L) (Figure 2C), which is expressed on mature DCs, activated B and T lymphocytes, and certain hematologic malignancies. This interaction provides a crucial co-stimulatory signal, enhancing T- and B-cell responses [127]. Upon engagement by CD70, CD27 activates NFκB (Figure 2C), promoting cell survival, enhancing proliferative signals mediated by T- and B-cell receptors, and boosting the cytotoxic T lymphocyte (CTL) response. This process supports the generation of immunological memory. Additionally, CD27 signaling reduces FAS ligand (FAS-L)-mediated T-cell apoptosis by inducing the expression of BCL-xL (Figure 2C) and PIM-1, thereby limiting mitochondrial dysfunction [37].

However, under specific circumstances, like in the chronically inflamed TME, the high levels of IL-2 secreted by CD70^+^CD4^+^ TILs elicit CD27 expression on Tregs, leading to improved Treg survival, ultimately inducing impairment of effector T-cell functionality [127].

Preclinical studies have reported that co-stimulatory molecule agonists can improve anti-PD-1/PD-L1 efficacy. In particular, the employment of an agonist anti-CD27 Ab has been reported to modestly improve an already effective approach combining a vaccine with anti-PD-1 therapy in ovarian cancer [128]. Accordingly, combining PD-1/PD-L1 blockade with an anti-CD27 agonist synergistically enhances CD8^+^ T-cell expansion and effector function. This effect is driven by IL-2 and c-MYC, with anti-PD-1/PD-L1 distinctly activating T-cell cytotoxicity, while anti-CD27 preferentially promotes T-cell proliferation. Additionally, the clinically relevant anti-human CD27 mAb, varlilumab (Figure 2C), has shown similar synergistic effects with PD-L1 blockade in protecting against lymphoma in human-CD27 transgenic mice, suggesting that the suboptimal T-cell activation observed in cancer patients treated with PD-1 blockade can be improved by a dual approach including PD-1 blockade and CD27 agonism, and providing mechanistic insights into the cooperative nature of these approaches in activating CD8^+^ T cells [129].

### 2.8. The ADA/CD26/A2AR Axis

Adenosine is a purine nucleoside that accumulates in the TME under stress conditions such as hypoxia and inflammation [130]. Elevated adenosine levels in the TME suppress immune cell function, thereby facilitating tumor immune evasion. Tumor cells, immune cells, and stromal cells can release ATP, which is converted to adenosine by ectonucleotidases like CD39 and CD73 [131]. The co-expression of CD39 and CD73 ensures the breakdown of ADP/ATP to AMP, followed by the transformation of AMP into adenosine (Figure 2C), ultimately shifting the local TME toward an immunosuppressive state [132], while serving as a marker for a regulatory phenotype [133].

Recent studies have identified CD73 within extracellular exosomes, providing a source of ectonuclease that can cooperate with T-cell-derived CD39 to increase local adenosine levels [134]. These findings are reinforced by a strong correlation between CD39 expression and local adenosine concentrations, highlighting the biological role of CD39-expressing T-cell subsets in enhancing adenosine levels, particularly with the involvement of extracellular CD73. Conversely, CD26 (also known as DPPIV), a protein involved in T-cell co-stimulation, proteolysis, and interaction with extracellular matrix proteins, has emerged as a negative selection marker for Tregs [135]. CD26^high^ CD4^+^ T cells also exhibit stem-like properties, enhanced intra-tumor migration, and stable memory capacities, enabling their persistence and activity against various solid tumors [136].

Adenosine deaminase (ADA), an enzyme that metabolizes adenosine into inosine, plays a critical role in modulating immune responses by either lowering local adenosine levels or acting as a co-stimulatory molecule through its interaction with CD26 (Figure 2C) [137,138]. A deficiency in ADA can lead to the accumulation of adenosine, promoting immunosuppression and allowing tumors to evade immune surveillance. Research indicates that overexpression of ADA enhances the antitumor efficacy of chimeric antigen receptor (CAR) T-cells in preclinical models [139]. Additionally, CD26 has emerged as a potential biomarker for response to PD-1 blockade [140]. Notably, a positive impact of the co-stimulatory ADA/CD26 axis has been recently reported on the functionality of CD8^+^PD-1^+^CD28^+^ T cells [43] in the immunosuppressive, CD39-enriched microenvironment of NSCLC [141], correlating with improved responses to ICB [43]. The critical role of CD28 in this context underscores the complex interactions and finely tuned balances that dictate immune responses and ICB efficacy in cancer patients.

In the TME, extracellular adenosine impairs CD8^+^ T-cell functionality primarily by engaging the A2A receptor (A2AR) (Figure 2C), a G protein-coupled receptor expressed on various immune cells, including T cells, NK cells, and macrophages. Engagement of adenosine to the A2AR activates inhibitory signaling pathways, impairing T-cell and NK cell activation and effector functions [142]. Given the role of A2AR in suppressing immune function and the role of CD39 and CD73 in generating immunosuppressive adenosine, targeting CD39, CD73, and A2AR is a promising strategy for manipulating antitumor immunity. In recent years, mAbs or small molecule inhibitors targeting the CD39/CD73/A2AR pathway have been investigated in clinical trials as single agents or in combination with anti-PD-1/PD-L1 therapies, recently reported by Xia et al. [143].

Targeting different components of this pathway, particularly A2AR and CD73, has shown potential in cancer therapy. For instance, the combination of sodium polyoxotungstate (a CD73 inhibitor) and AZD4635 (an A2AR antagonist) has been reported to impair adenosine production, leading to enhanced immune activity, increased INF-γ production, and reduced Treg frequency [144]. Combining anti-CD39 and anti-CD73 Abs has been reported to contrast T-cell inhibition in myeloma models. Clinical trials involving combinations of A2AR inhibitors with ICIs, like CPI-444 with atezolizumab, have shown stronger antitumor responses than single-agent treatments, particularly in NSCLC and prostate cancer [145].

Other studies have investigated a combination of CD39/CD73/A2AR inhibitors with conventional cancer therapies. For example, combining an anti-CD73 mAb with chemo-photodynamic therapy has demonstrated enhanced antimetastatic effects in animal models of triple-negative breast cancer by boosting T-cell-mediated immunity while preventing abscopal effect [146], involved in tumor metastasis [147] by inducing systemic cytotoxic T-cell response via CD73 blockade [146]. Dose-limiting toxicities were noted in some clinical trials, such as with IPH5301 and trastuzumab. Blocking the adenosine pathway can also improve responses to other therapies, like NK cell treatments and DC-based vaccines, by increasing cytotoxic T-cell infiltration and antitumor immune responses [146].

However, although this pathway remains a promising target for enhancing cancer immunotherapy, more research is needed to optimize combinations and minimize toxicity.

Moreover, not all tumors exhibit elevated adenosine levels in the TME. Due to the rapid metabolism of adenosine, which has a plasma half-life of only about 10 s [148], direct quantification of its levels in patient samples is challenging [149]. This makes adenosine an impractical biomarker for assessing tumors through direct measurement, and patient selection for A2AR-targeting therapies is critical. Identifying biomarkers able to identify tumors with high adenosine and A2AR expression could improve the efficacy of these treatments.

Gene expression profiles related to adenosine have been found to strongly correlate with its levels in tumors, offering an alternative approach. These adenosine-related gene signatures may serve as predictive biomarkers for responses to treatments targeting the adenosine-A2AR pathway. Indeed, a recent study has identified a set of genes involved in myeloid cell biology and inflammation that are positively correlated with adenosine levels. This has led to the development of the “Adenosine Gene Signature” (AdenoSig), a group of eight genes (*CXCL1*, *CXCL2*, *CXCL3*, *CXCL5*, *CXCL6*, *CXCL8*, *PTGS2*, *IL-1β*) [150], used to identify patients likely to respond to the A2AR antagonist ciforadenant. Additionally, Sidders et al. have proposed another genomic profile, the “Adenosine Signaling Score”, comprising 14 genes (including *PPARG*, *CYBB*, *FOXP3*, and *LAG3*), which is closely linked to A2AR signaling in various cancers, thus potentially helping to predict responses to immunotherapy [151]. These gene signatures provide promising tools for tailoring treatment strategies in cancer therapy. Notably, enhancing ADA activity could also help reverse this immunosuppressive environment.

## 3. Overview of the Inhibition of Immune Responses by ICs

ICs are crucial in regulating the immune system and preventing excessive immune responses that could lead to tissue damage. However, tumors often exploit these checkpoints to evade immune surveillance, leading to immune tolerance and tumor progression. In recent years, ICIs have emerged as an invaluable approach to cancer immunotherapy.

ICIs block the interaction between checkpoint molecules and their ligands, releasing the brakes on the immune system and unleashing antitumor immune responses. The clinical success of ICIs has revolutionized cancer treatment across various malignancies, including melanoma, NSCLC, renal cell carcinoma, and bladder cancer [152]. ICIs have shown durable responses and improved OS in patients with advanced or metastatic disease. Additionally, combination therapies involving dual CB or ICIs combined with other treatment modalities such as chemotherapy, targeted therapy, or radiation therapy have demonstrated synergistic effects and improved clinical outcomes.

Despite their remarkable efficacy, only about 20–30% of patients respond to ICIs, necessitating the implementation of patient stratification to identify potential responders and a deeper understanding of the underlying mechanisms. Ongoing research aims to optimize treatment strategies, identify predictive biomarkers of response, and develop novel combination therapies to further enhance the efficacy of immunotherapy and expand its application across a broader spectrum of malignancies. Herein, the key concepts of immune inhibition by ICs and their main cross-talks are discussed.

### 3.1. CTLA-4 Mediated Crosstalk: CD28, CD80, and Beyond

**Figure 3:**
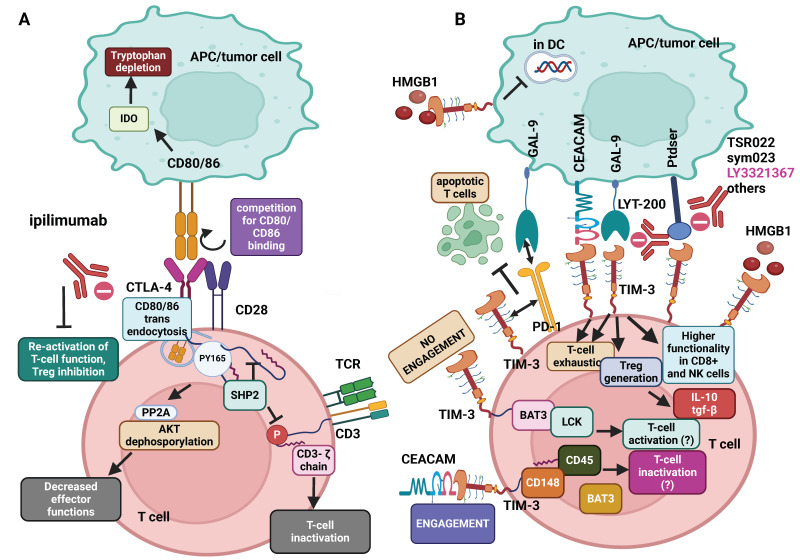
Selected aspects of the complex roles and interactions of inhibitory molecules CTLA-4 and TIM-3 in regulating T-cell activation within the TME, as described in the main text. Main ligands and intracellular signals are depicted, highlighting their roles in controlling antitumor immune response. (**A**) CTLA-4 blocks CD28-mediated co-stimulation by competing for the binding to CD80/86 and leading to their degradation via trans-endocytosis. CTLA-4 also induces IDO in APCs, resulting in localized tryptophan depletion, suppression of effector T cells, and generation of Tregs. Ipilimumab, blocking CTLA-4, enhances CD28 co-stimulation. Phosphorylation of CTLA-4 cytoplasmic tail at Y165 disrupts its interaction with PP2A, allowing its binding to AKT, leading to its deactivation; (**B**) TIM-3 binds to GAL-9, CEACAM1, Ptdser, and HMGB1. Engagement of TIM-3 with GAL-9 and CEACAM impairs T-cell functionality and induces T-cell exhaustion. TIM-3/GAL-9 engagement induces Tregs by promoting IL-10 and TGF-β release. Conversely, in CD8^+^ T cells and innate immunity cells, the TIM-3/GAL-9 interaction can have stimulatory activity. An interesting model proposes that in the absence of ligand binding, TIM-3 associates with BAT3, recruiting active LCK to promote T-cell activation, while upon ligand engagement, BAT3 dissociates, allowing TIM-3 to exert its inhibitory effects. PD-1 can physically interact with both GAL-9 and TIM-3, dampening GAL-9/TIM-3-induced T-cell apoptosis. HMGB1 binding to TIM-3 on DCs impairs the transport of tumor-derived nucleic acids to the endosome. Different anti-TIM-3 mAbs (TSR022, Sym023, LY3321367) block mainly the TIM-3/Ptdser engagement site. LYT-200 targets engagement with GAL-9. The red circle with a minus sign represents inhibition of receptor activity and of its interaction with the ligand, the green circle with a plus sign indicates receptor activation. The pointed arrow indicates activation, the flat-tipped arrow indicates inhibition. The question mark (?) indicates a possible mechanism. Created in BioRender. Franzese, O. (2024) https://BioRender.com/u78h488 (accessed 22 November 2024).

CTLA-4, a glycoprotein of the Ig superfamily, resembles CD28 co-stimulatory molecule by engaging with oligomerized forms of CD80 and CD86 ligands (Figure 3A) but instead delivering inhibitory signals. CTLA-4 interferes with T-cell activation by competing with CD28 for the binding to CD80/86, thus blocking CD28-mediated co-stimulation and hampering IL-2 secretion, while cells expressing CTLA-4 capture CD80/86 molecules, leading to their degradation via trans-endocytosis (Figure 3A) [153]. Therefore, the balance between B7 molecules binding to CD28 or CTLA-4 dictates the T-cell activation status. CTLA-4 triggers additional inhibitory signals via CD80/CD86, inducing indoleamine 2,3-dioxygenase (IDO) expression in APCs, resulting in localized tryptophan depletion (Figure 3A), suppression of effector T cells, and generation of Tregs. The role of CTLA-4 in the generation of Tregs is critical in regulating the immune response and disease therapy. Indeed, deleting CTLA-4 in Tregs leads to widespread lymphocyte proliferation, fatal autoimmune diseases, heightened IgE production in mice, and robust tumor immunity [153].

CTLA-4 blockade with ipilimumab and other Abs has been reported to impede tumors through various mechanisms, primarily by favoring CD28 binding to CD80/86, especially in tumor-draining lymph nodes where APCs cross-present tumor antigens to activate tumor-reactive T-cells. Effective antigen presentation, facilitated by CTLA-4 blockade, enhances CD28 co-stimulation, activating the T-cell immune response [154]. Regulation of the TCR repertoire may also contribute to the therapeutic effects of CTLA-4 inhibition [13]. CTLA-4 absence or blockade may lower the threshold of TCR ligation required for T-cell activation, boosting T-cell stimulation in multiple ways and fostering more active tumor-reactive T-cells. Upon TCR engagement, CTLA-4 expression rises, and intracellular vesicles containing CTLA-4 migrate to the immune synapse. Phosphorylation of CTLA-4 cytoplasmic tail at Y165 (Figure 3A) by TCR-induced kinases disrupts its interaction with PP2A phosphatase, maintaining cell-surface CTLA-4 levels in the immune synapse. Strong TCR signals lead to increased accumulation of CTLA-4 in the immune synapse, offering a modulable inhibitory signal. Although involving AKT deactivation, CTLA-4-mediated negative regulation of T-cell function does not rely on PI3K inactivation [15,155]. The interactions between CTLA-4 and the serine/threonine phosphatase PP2A have been independently confirmed [156]. When TCR and CTLA-4 are co-ligated, CTLA-4 binds to B7 molecules on APCs. This binding causes PP2A to detach from CTLA-4 to bind to AKT, leading to its deactivation. As a result, this process inhibits AKT activity and reduces T-cell activation (Figure 3A) [155]. Additionally, CTLA-4 has been found to bind to the tyrosine phosphatase SHP-2. Activation of SHP-2 suppresses the CD3/CD28-induced T-cell transcriptional profile and leads to the inactivation of LCK and ZAP-70 kinases as well as dephosphorylation of the CD3-ζ chains of the TCR complex (Figure 3A), ultimately reducing TCR signaling activation. However, the exact role of SHP-2 in the CTLA-4-associated negative regulation of T-cell function remains incompletely understood.

### 3.2. PD-1 and CD28: A Dynamic Interplay in T-Cell Activation

PD-1, first identified in 1992 as a potential mediator of apoptosis [157], has since been established as a critical IC within the B7-CD28-CTLA4 family, crucial for preventing overactivation of the immune system [158]. It plays a vital role in maintaining peripheral tolerance and modulating T-cell responses through its interactions with the ligands PD-L1 and PD-L2, thereby influencing T-cell activation and inducing T-cell exhaustion [159].

PD-L1 is primarily expressed on APCs in response to pro-inflammatory cytokines, such as IFN-γ (Figure 1A) [160]. However, it can also be found in non-hematopoietic tissues under normal conditions. Of note, a PD-L1/CD80 cis-heterodimerization has been reported on APCs, able to preserve the CD80 capacity to activate CD28, thereby supporting the sustained functionality of intra-tumor CD28^+^ T cells while repressing the inhibitory activity of PD-1 and CTLA-4 (Figure 1A) [161].

Upon TCR stimulation, PD-1 undergoes phosphorylation at specific tyrosine residues adjacent to the immunoreceptor tyrosine-based inhibition motif (ITIM) and the immunoreceptor tyrosine-based switch motif (ITSM) on its cytoplasmic tail. This phosphorylation initiates the recruitment of phosphatases SHP-1 and SHP-2 (Figure 1A), which dephosphorylate downstream signaling molecules of the TCR and CD28, inhibiting proximal TCR signals at immune synapses.

Moreover, PD-1 inhibits AKT-mediated T-cell activation (Figure 1A) and impairs CD28-mediated glucose uptake by disrupting proximal PI3K signaling through SHP-2 [155]. Thus, the combined actions of CTLA-4 and PD-1 activate partially overlapping but distinct intracellular signaling pathways.

Recent studies have shown that SHP2 engagement with PD-1 dephosphorylates the γc chain at tyrosine 357, which weakens the γc-mediated signaling, dampening immune activation [162]. In the absence of SHP-2, PD-1-induced dephosphorylation of γYc357 is impaired, leading to increased phosphorylation of γYc357 in response to the cytokines transmitting their signal through the γc family.

In maintaining immune balance, PD-1 induces Tregs, establishing a stringent activation threshold for T cells and preventing autoimmunity [158]. Blocking PD-1 signaling can enhance tumor rejection by reinvigorating CD8^+^ T cells, thereby improving their functionality and frequency. This reinvigoration is supported by metabolic reprogramming induced by the disruption of PD-1 signaling [158].

Nivolumab, the first approved anti-PD-1 mAb, showed promising results in Phase I trials for metastatic melanoma, NSCLC, and renal cell cancer. Pembrolizumab, a highly selective PD-1 targeting mAb, has paved the way for the development of similar Abs, including pidilizumab, MPDL3280A, targeting PD-L1 [163], BMS-936559 [164], and MEDI4736 (durvalumab) [165]. Among the newer Abs targeting the PD-1/PD-L1 axis, cemiplimab, toripalimab, cindilimab, camrelizumab, tislelizumab, and dostarlimab represent just a fraction of those currently available, as reviewed by Liu et al. [166].

### 3.3. TIM-3 and the Cross-Talk with Its Ligands

TIM-3 significantly impacts CD8^+^ T-cell exhaustion in cancer [167]. Upon binding to soluble GAL-9 [168], Carcinoembryonic Antigen-related Cell Adhesion molecule 1 (CEACAM1) [169], Phosphatidylserine (Ptdser) [170], or the alarmin High Mobility Group Box 1 (HMGB1) (Figure 3B) [171], TIM-3 contributes to immune tolerance and other crucial regulatory functions. GAL-9 belongs to the GAL family, a group of animal lectins characterized by Conserved Carbohydrate-Recognition Domains [CRDs], specific for β-galactosides [172]. Its unique structure enables GAL-9 to generate multimeric complexes by crosslinking glycoproteins, generating GAL-glycoprotein lattices crucial for regulating various cellular processes. GAL-9 interacts with the N-linked glycans located within the IgV domain of TIM-3, whereas other ligands engage with a distinct site known as the FG-CC loop within the same domain [173]. Different anti-TIM-3 mAbs (TSR022, Sym023, ICAGN02390, BGB-A425, and MBG453) blocking mainly the TIM-3/Ptdser engagement site are investigated in clinical trials. Among them, only LY3321367 partially blocks the TIM-3/GAL-9 interaction [174]. Of note, LYT-200, a human IgG4 mAb targeting GAL-9 (Figure 3B), that has been reported to inhibit tumor growth in mice models, improve survival, and enhance the quality of the immune environment [175], is currently in an adaptive Phase I/II clinical trial (NCT04666688, https://clinicaltrials.gov/, last accessed 31 October 2024) for relapsed or refractory solid tumors.

Through its engagement with TIM-3, GAL-9 impairs Th1-mediated immunity [168] and plays a pivotal role in modulating TIM-3-mediated T-cell exhaustion (Figure 3B) and death of TIM-3^+^ T cells [176].

PtdSer and HMGB1 bind to distinct regions on the TIM-3 extracellular IgV domain and play a crucial role also in antigen cross-presentation by TIM-3^+^ DCs, affecting innate immune activation and potentially impacting disease progression, as reported for colon carcinoma models. Notably, the interaction of CEACAM1 with TIM-3 expressed by T cells has been reported to impair T-cell functionality while contributing to immune tolerance (Figure 3B) [169]. Concerning the outcomes of the interactions of TIM-3 with GAL-9 and CEACAM1, observations indicate that, despite their binding to separate regions within the IgV domain, they elicit similar downstream signaling cascades [177].

Although the interaction between TIM-3 and HMGB1 is not well understood, it has been reported that HMGB1 binding to TIM-3 on DCs impairs the transport of tumor-derived nucleic acids to the endosome (Figure 3B), thereby inhibiting the pattern recognition receptor (PRR)-mediated immune response [178].

GAL-9 exhibits critical roles in T-cell apoptosis and homeostasis, production of cytokines, and differentiation of Tregs, highlighting GAL-9 as a critical regulator of immune responses [179]. Notably, the TIM-3/GAL-9 engagement supports the immunosuppressive role of tumor-associated Tregs by promoting the release of IL-10 and TGF-β (Figure 3B).

Studies indicate that PD-1 and TIM-3 are co-expressed in TILs exhibiting an exhausted phenotype and characterized by reduced secretion of IFN-γ, IL-2, and TNF-α [179,180], suggesting that a synergistic effect can be achieved by inhibiting both checkpoints.

However, the impact of the GAL-9/TIM-3 axis depends on TME features and the specific immune cells involved. In normal physiological conditions, TIM-3 is expressed on T-cell subsets (Th1, Th17, and Tc1) and macrophages, exerting inhibitory effects upon binding with GAL-9. Conversely, in CD8^+^ T cells and innate immunity, the TIM-3/GAL-9 interaction has been reported to have stimulatory activity (Figure 3B) [181,182].

Some confounding findings have also been reported concerning the TIM-3/GAL-9 pathway involvement in tumor pathogenesis. Early studies described lower GAL-9 expression in metastatic melanoma lesions than in primary lesions [183]. Moreover, the ectopic expression of GAL-9 has been reported to inhibit metastasis in GAL-9-deficient B16F10 mouse melanoma cells [184]. Differently, more recent studies have reported a positive involvement of GAL-9 in metastasis when CCR7 is highly expressed in metastases from melanoma patients, also co-expressing PD-L1 [185]. In colon cancer, high GAL-9 expression induces apoptosis in cancer cells and has been linked to better OS [186]. In breast cancer, while early studies reported an anti-metastatic role [187], more recent investigations have described that GAL-9 expressed in tumor cells can drive tumor invasiveness and affect chemotherapy outcomes [188].

In the context of glioma, both TIM-3 and GAL-9 are expressed at significantly higher levels than in healthy brain cells. Elevated TIM-3 expression correlates with World Health Organization (WHO) grades II to IV gliomas, indicating disease progression. As the severity of gliomas increases from Grade II/III to Grade IV, there is a corresponding rise in TIM-3 expression on CD4^+^ and CD8^+^ T cells and increased GAL-9 expression within tumors. Therefore, the impact of the GAL-9/TIM-3 axis depends on TME features and the specific immune cells involved, and additional studies are needed to unveil the complete molecular mechanism of the TIM-3/GAL-9 pathway in order to broaden its therapeutic applications for cancer therapy. Additionally, a specific subset of tumor-infiltrating immune cells responsive to GAL-9 blockade has been identified, leveraging Authors to suggest that a combinatorial therapeutic approach able to boost the antitumor efficacy of anti-GAL-9 therapy could be applied [172]. Remarkably, perturbation of the TIM-3/GAL-9 pathway has been observed in various cancers, with studies showing increased levels of TIM-3 and GAL-9 in cancers including Acute Myeloid Leukemia (AML), prostate cancer, NSCLC, esophageal squamous cell carcinoma (ESCC), breast cancer, glioma, head and neck cancer, colon cancer, melanoma, and lung adenocarcinoma [174].

A T-cell stimulatory role has also been proposed for TIM-3, primarily stemming from in vitro investigations, showing an enhanced TCR signaling in Jurkat T cells, leading to improved NFAT/AP-1 activation [189], an observation that has contributed to sparking a debate over whether TIM-3 can also act as a co-stimulatory receptor.

Interestingly, increased TIM-3 expression has been linked to adaptive resistance to PD-1 blockade. In murine models of HNSCC, TIM-3 expression is upregulated in response to PD-1 inhibition, driven by the activation of the PI3K/AKT/mTOR pathway via cross-talk between PD-1 and TIM-3 [190,191]. This highlights a potential interplay between TIM-3, its ligands, and downstream signaling molecules, which may influence T-cell function and immune responses.

Indeed, TIM-3 regulatory mechanisms remain largely undefined. Unlike PD-1, TIM-3 lacks recognized inhibitory signaling motifs, although Tyr256 and Tyr263 within its cytoplasmic tail facilitate interactions with BAT3 and the tyrosine kinase FYN [167]. An interesting model has proposed that in the absence of ligand binding, TIM-3 associates with BAT3, recruiting active LCK to promote T-cell activation (Figure 3B). At variance, upon ligand engagement, BAT3 dissociates, allowing TIM-3 to exert its inhibitory effects, possibly through interaction with receptor phosphatases such as CD45 and CD148 [167]. This suggests a complex regulatory mechanism involving TIM-3, CEACAM1, and GAL-9 within the TME. Co-expression of TIM-3 and PD-1 is observed in multiple cancers, suggesting a potential target for combination therapy. For instance, in lung adenocarcinoma, high TIM-3 levels in TILs and GAL-9 on KRAS-mutant tumors are associated with resistance to PD-1 blockade. In advanced melanoma, TIM-3^+^PD-1^+^CD8^+^ T cells exhibit significant T-cell suppression. Similarly, in hepatocellular carcinoma (HCC), TIM-3 expression on T cells and GAL-9 on Kupffer cells has been shown to impair effector T-cell function [174,192].

It is well known that the co-expression of PD-1 and TIM-3 characterizes a highly exhausted T-cell population within tumors [176] by exhibiting reduced long-term survival compared to precursor-exhausted T cells expressing only PD-1. Remarkably, since 2016, eight anti-TIM-3 or PD-1/TIM-3 bispecific Abs have been tested in clinical trials. TSR-022, the first anti-TIM-3 Ab examined, showed preliminary efficacy and safety, with higher adverse event rates when combined with anti-PD-1 blockade. LY3415244 was terminated due to poor risk/benefit, while LY3321367 continues with promising early results. MBG453 demonstrated safety and efficacy in acute myeloid leukemia and solid tumors. Sym023 showed safety in patients with advanced solid tumors, and other anti-TIM3 Abs are still under investigation in Phase I trials [193].

Interestingly, PD-1^+^TIM-3^+^ T cells seem to endure within the TME, often dominating the tumor-infiltrating CD8^+^ T-cell population in specific cancers. Their presence is related to tumor reactivity and has been involved in predicting response to PD-1 blockade. A notable study by Yang et al. [194] has investigated the molecular mechanisms underlying the persistence of exhausted T cells within the TME, showing that PD-1 physically interacts with both GAL-9 and TIM-3, thereby dampening GAL-9/TIM-3-induced T-cell apoptosis (Figure 3B). These observations reveal a novel role for PD-1 in modulating exhausted T-cell survival and propose GAL-9 as a critical regulator of the tumor immune response and a promising target for cancer immunotherapy strategies.

Targeting the TIM-3-CEACAM1 axis in cancer immunotherapy also holds promise. As discussed by Sakuishi et al. [176], given the diverse array of TIM-3 ligands and the unknown affinity of TIM-3 for each of them, it is plausible that their distribution and expression levels within the TME dictate the signaling pathways activated by TIM-3, suggesting that different networks and cross-talks may occur in different environments.

### 3.4. The Cross-Talk Between the TIGIT/CD226/CD155 and the PVRIG Axes

**Figure 4:**
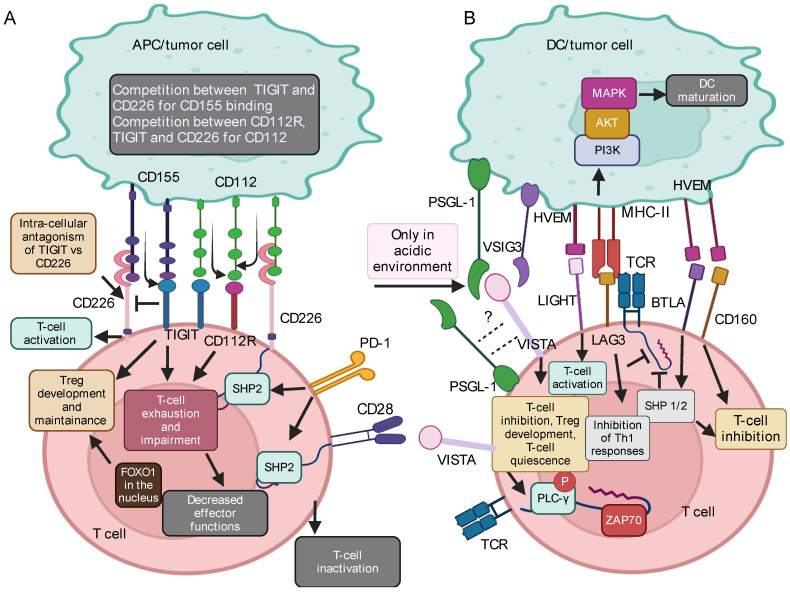
Selected aspects of the complex roles and interactions of the TIGIT/CD226/CD155 and PVRIG axes, along with the inhibitory molecules LAG-3, BTLA, and VISTA, in regulating T-cell activation within the TME are illustrated, as described in the main text. Main ligands and intracellular signals are depicted, highlighting their roles in controlling antitumor immune response. (**A**) TIGIT interacts with CD155 and CD112, among other ligands. TIGIT^+^ Tregs exhibit consistent nuclear FOXO1 localization, enhancing their suppressive capabilities. The immunomodulatory effects of TIGIT depend on CD226, which is inhibited by TIGIT through intra-cellular antagonism and by the competition for the binding to CD155 and CD112. CD226 and CD112R also compete with TIGIT for the binding to CD112. Binding affinities of CD155 to TIGIT and of CD112 to CD112R are higher than those of CD226, indicating that TIGIT and CD112R may outcompete CD226 for ligand binding. While TIGIT delivers an immunosuppressive signal, the engagement of CD226 to either CD155 or CD112 provides co-stimulation. Similarly to the PD-1/CD28 axis, TIGIT dampens activating signals through CD226; (**B**) LAG-3 selectively binds stable peptide-MHC II complexes and inhibits activation, proliferation, and cytokine secretion of Th1 cells. The inhibitory function of BTLA is primarily mediated through its interaction with HVEM, which recruits protein tyrosine phosphatases SHP-1 and SHP-2. Interactions between HVEM on DCs or Tregs and either BTLA or CD160 trigger inhibitory signaling pathways, suppressing T-cell activation, while HVEM engagement with LIGHT on T cells provides a stimulatory signal. VISTA is especially prevalent in FOXP3^+^ CD4^+^ Tregs, contributing to promoting their differentiation and maintaining their phenotype while suppressing effector T-cell function. VISTA interacts with two key ligands, VSIG3 and PSGL-1, and binds to PSGL-1 exclusively at the acidic pH. The pointed arrow indicates activation, the flat-tipped arrow indicates inhibition. Created in BioRender. Franzese, O. (2024) https://BioRender.com/f91v568 (accessed 22 November 2024).

TIGIT, playing a crucial role in immune regulation [195], belongs to the poliovirus receptor (PVR)/nectin family, which includes, among others, PVR (CD155) and PVRL2 (CD112 or Nectin-2). Recent reviews, including the one recently provided by Zhang et al., highlight the structure, function, and ligands of TIGIT [196]. TIGIT is predominantly expressed on CD8^+^ and CD4^+^ TILs, Tregs, and NK cells, with intra-tumoral TILs showing higher levels than their circulating counterparts [44]. TIGIT^+^ Tregs exhibit consistent FOXP3 activity and nuclear FOXO1 localization, enhancing their suppressive capabilities. Inhibition of the PI3K/AKT/mTOR pathway preserves Treg identity by maintaining FOXO1 in the nucleus (Figure 4A) [197]. Interestingly, a distinct role for TIGIT has been suggested in both solid and hematological tumors, as demonstrated by its high expression on CD4^+^ but not CD8^+^ T cells in chronic lymphocytic leukemia (CLL) [198].

The expression of TIGIT can be modulated by type 1 IFN and chemotherapy, while factors like microwave ablation, glucose deprivation, and hypoxic conditions can trigger its expression in tumor cells [196]. TIGIT interacts with four ligands, including nectin and nectin-like adhesion molecules: CD155, CD112 (Figure 4A), CD113 (PVRL3, Nectin-3), and nectin-4 (PRR4, PVRL4). TIGIT binds with the highest affinity CD155, which serves as its primary ligand [199].

Notably, it has been reported that the immunomodulatory effects of TIGIT depend on CD226, which is inhibited by TIGIT through intra-cellular antagonism and by the competition for binding to CD155 and CD112 (Figure 4A). Notably, while TIGIT delivers an immunosuppressive signal, the engagement of CD226 to either CD155 or CD112 provides co-stimulation. Therefore, this competition can potentially fuel activating signals in T cells, promoting cytotoxicity and cytokine production against cancer cells (Figure 4A) [196,199,200]. The CD226 receptor exhibits significant analogies with CD28. Similarly to the PD-1/CD28 axis, TIGIT expression is up-regulated following T-cell activation, dampening activating signals through CD226. Instead, similarly to the CTLA-4/CD28 axis, TIGIT can outcompete CD226 for engagement with their common ligands CD155 and CD112 [200,201].

Recent evidence indicates that the inhibition of co-stimulatory molecules is more intricate than initially understood. Both CD28 and CD226 are regulated not only by their respective inhibitory receptors (CTLA-4, PD-1, and TIGIT) but may also be dephosphorylated by the SHP2 phosphatase (Figure 4A), which is recruited to the PD-1 intracellular domain following T-cell activation and phosphorylation by LCK [40,201,202].

Notably, in NSCLC patients treated with atezolizumab, the expression of CD226 has been reported to be directly linked to significant clinical benefits. Indeed, since PD-1 blocks the phosphorylation of both CD226 and CD28, dual inhibition of TIGIT and PD-1 is required to fully restore CD226 signaling and optimize CD8^+^ T-cell responses, supporting the rationale for combined therapy [202]. Thus, these two inhibitory receptors are interdependent, suggesting that their coordinated inhibition is required to elicit optimal T-cell activity. Further investigation has revealed the highest CD226 and CD28 co-expression in TME-derived memory CD8^+^ T-cell subsets with high capacity for self-renewal and the potential to differentiate into memory and effector cells [202], a population likely re-invigorated by ICB.

In summary, the inhibitory function of TIGIT relies on a delicate balance between its interaction with CD155 and CD112 on cancer cells, the expression levels of CD226 on T cells, and the co-presence of PD-1. Therefore, targeting CD226 has potential therapeutic benefits, but challenges result from the complexity of this signaling axis. Accordingly, recent findings suggest that the effectiveness of TIGIT inhibition may be enhanced by combination with CD226-targeted therapies. These observations relate to the overlapping yet distinct functions of TIGIT and CD226, which can influence immune responses [202]. Preclinical data indicate that the inhibition of TIGIT combined with CD226 stimulation could overcome limitations and enhance antitumor activity. Given the potential for compensatory mechanisms within the CD226 axis, incorporating TIGIT blockade into treatment strategies could maximize the therapeutic potential of ICIs. The CD226-targeting agent LY3435151 has advanced to Phase I clinical study (NCT04099277), which was terminated shortly after it began. Nevertheless, the important role of CD226 in the CD155 and CD112 pathways strongly suggests that understanding these limitations will be crucial for future developments [201,202].

An intricated cross-talk exists between the above-described TIGIT/CD226/CD155 pathway and the CD112-PVRIG (CD112R) axis, highlighting a multifaceted and intricate immunoregulatory mechanism that has only recently been uncovered [200,203]. The PVR-like molecules belong to the Ig superfamily and play critical regulatory roles in T-cell and NK cell functions. Their ligands are expressed on tumor cells, APCs, and endothelial cells, contributing to key immunoregulatory mechanisms in tumor progression [203].

PVRIG, also known as CD112R, was identified in 2016 as a novel IC receptor [204], expressed on T and NK cells, which exerts an immunosuppressive effect by specifically binding to its ligand CD112.

As previously mentioned, CD112 can also bind to TIGIT and CD226. TIGIT competes with CD226 for the binding to CD155 [199], while CD226 and CD112R compete with TIGIT for the binding to CD112 (Figure 4A). Therefore, CD112R exerts its inhibitory effects through a distinct regulatory pathway, which intersects with CD226 to modulate immune responses, leading to variable activation outcomes depending on which receptor predominates. Functionally, the engagement of CD112 with either CD112R or TIGIT impairs antitumor immune responses, whereas its interaction with CD226 enhances antitumor immunity by boosting T and NK cell cytotoxicity [205]. Of note, the binding affinities of CD155 to TIGIT and CD112 to CD112R are higher than those of CD226, indicating that TIGIT and CD112R may outcompete CD226 for ligand binding (Figure 4A) [206].

A recent study by Stamm et al. [207] has demonstrated that mAbs targeting either CD155 or TIGIT enhance the killing of breast cancer cell lines by cytokine-induced killer cells. Given that CD112R competes with both TIGIT and CD226 for CD112 binding, blocking both the CD112R/CD112 and TIGIT/CD155 axes could allow CD226 unrestricted access to its ligands.

CD112R expression is found on TILs and intratumoral NK cells. In vitro studies have shown that co-targeting CD112R and TIGIT with mAbs enhances the effector functions of human TILs and NK cells. Additionally, combining anti-CD112R with PD-L1 blockade has been shown to reduce tumor growth, confirming the outcomes observed in CD112R-deficient mice treated with PD-L1 blockade. These observations suggest the co-targeting of CD112R and TIGIT checkpoints as a promising strategy for improving antitumor immune responses [208].

Results from a Phase I safety and preliminary efficacy trial of PM1009, a bispecific antibody targeting TIGIT and CD112R in patients with advanced solid tumors, presented at the 2024 ASCO Annual Meeting, have reported good tolerability and early signs of antitumor activity, thus supporting further clinical investigation, particularly in combination with chemotherapy or PD-1/L1 blockade [209]. Overall, detailed characterization of the intra-tumor expression of CD226, CD155, TIGIT, CD112, and CD112R can provide critical insights into the tumor immune landscape, supporting patient selection for more effective, tailored immunotherapeutic strategies.

### 3.5. LAG-3

LAG-3 (CD223) is a type I membrane protein consisting of distinct cytoplasmic, transmembrane, and extracellular regions with a hydrophobic leader. Its extracellular portion is structured into four domains resembling the Ig superfamily, with the membrane-distal domain containing a unique “extra loop” amino acid sequence [210,211]. Within the cytoplasmic domain, the conserved regions identified include glutamic acid–proline repeats and a serine phosphorylation KIEELE motif site, the latter crucial for LAG-3 inhibitory function. Metalloproteases cleaving LAG-3 within the transmembrane domain produce a soluble form, sLAG-3, which dampens T-cell immunological responses [211]. Beyond plasmacytoid DCs, LAG-3 is prevalent in activated CD4^+^ and CD8^+^ T cells, Tregs, a subset of NK cells, and B cells. Evidence suggests that LAG-3 signaling negatively regulates the activation, proliferation, and cytokine secretion of Th1 cells, mechanisms exploited by tumor cells to evade immune surveillance during carcinogenesis and metastasis.

Although most studies on LAG-3 have primarily focused on its role in T-cell dysfunction (Figure 4B), the impact of LAG-3 in the TME extends beyond T cells, as it also influences other immune cells, such as DCs and NK cells [212], shaping the overall immune response to tumors. While the physiological function of LAG-3 remains incompletely understood, inhibitors targeting this molecule have demonstrated promising therapeutic potential. Several anti-LAG-3 mAbs are currently undergoing clinical trials, and the combination of relatlimab (anti-LAG-3) with nivolumab (anti-PD-1) has already been approved for treating unresectable or metastatic melanoma, based on results from the Phase II/III RELATIVITY-047 (NCT03470922) [213]. How these two inhibitory receptors synergize to hinder antitumor immunity has been partially described by Andrews et al. [214], who have shown how CD8^+^ T cells lacking both PD-1 and LAG-3 show enhanced tumor clearance and improved survival in mouse models of melanoma, outperforming cells deficient in either receptor alone. These dual-deficient T cells have broad TCR diversity, are enriched in effector-like and IFN-responsive genes, and release more IFN-γ, indicating greater functionality. PD-1 and LAG-3 jointly drive T-cell exhaustion, with a critical role in modulating TOX expression, highlighting the potential of targeting both PD-1 and LAG-3 for more effective cancer immunotherapy.

Despite these advances, the precise mechanisms underlying the LAG-3 effect in the tumor setting must still be elucidated.

A comprehensive systematic summary of LAG-3 structure, ligands, cell-specific functions, and the inhibitors currently under development has been recently provided by Luo et al. [215].

Structurally akin to CD4 [216], LAG-3 binds MHC-II, exhibiting a 100-fold higher binding affinity than CD4. LAG-3 selectively binds stable peptide-MHC II complexes (Figure 4B), suppressing T-cell responses by transmitting inhibitory signals. This interaction does not interfere with CD4-MHC II binding, but the mechanism suggests that LAG-3 preferentially inhibits T cells responsive to stable peptide-MHC II complexes. Multiple other proteins, including LSECtin and α-synuclein [217], have been identified as interacting partners with LAG-3. LAG-3 also affects CD8^+^ T cells independently of MHC II, leading to the exploration of other ligands. LSECtin, highly expressed in the liver and lymph nodes, interacts with LAG-3 to inhibit effector T-cell proliferation by down-regulating the cell cycle kinases (CDK2, CDK4, and CDK6) [218]. Notably, the interaction of LAG-3 with LSECtin, expressed in melanoma cells, leads to the inhibition of IFN-γ secretion by effector T cells, while GAL-3, another potential ligand, suppresses CD8^+^ T cells and DCs. FGL1, a fibrinogen-like protein, is another crucial immunosuppressive ligand of LAG-3, inhibiting T-cell activation and associated with poor prognosis in cancers like HCC. Lastly, LAG-3 also interacts with the TCR/CD3 complex, inhibiting TCR signaling in both CD4^+^ and CD8^+^ T cells, suggesting new therapeutic targets beyond the classical MHC II/LAG-3 pathway.

Notably, LAG-3, expressed on activated T cells, can bind to MHC II expressed by immature DCs, promoting their maturation [219]. This interaction triggers the production of cytokines like IL-12 and TNF-α [220], able to enhance T-cell proliferation and Th1 responses. Key downstream signaling pathways activated by LAG-3/MHC II include PI3K/AKT, ERK1/2, and p38 MAPK (Figure 4B), all of which contribute to DC maturation. At variance, soluble LAG-3 (sLAG-3) has been shown to reduce monocyte differentiation into macrophages and DCs, indicating a regulatory role in limiting T-cell responses [221]. Further research is needed to clarify how LAG-3 influences macrophage and DC production in vivo.

### 3.6. Focus on BTLA as an Inhibitory Molecule

BTLA, also known as CD272, is a member of the CD28 superfamily, sharing structural features with PD-1 and CTLA-4, which binds to HVEM, a member of the TNFR family [222]. The *BTLA* gene encodes a transmembrane glycosylated protein with three splicing variants and is predominantly expressed in B and T cells (Figure 4B), contributing to the inhibition of the activation and proliferation [100,222]. Additionally, BTLA is found in DCs, where it plays a role in regulating homeostasis, proliferation, and cytokine production. Notably, genetic variations in BTLA, particularly *rs1982809*, have been associated with increased susceptibility to various malignancies, including renal cell carcinoma, breast cancer, and lung cancer, especially among Caucasians.

The inhibitory function of BTLA is primarily initiated through its interaction with HVEM, which recruits protein tyrosine phosphatases SHP-1 and SHP-2 (Figure 4B), leading to immunosuppression by dephosphorylating TCR-activated tyrosine kinases. Initial assumptions suggested that both SHP-1 and SHP-2 were involved; however, later studies clarified that only SHP-1 is recruited, and mutations affecting this interaction do not impact BTLA function [222].

Interestingly, the YDND motif of BTLA, upon engagement, binds to GRB-2, facilitating PI3K recruitment and activating survival signals that lead to T-cell activation. Additionally, soluble BTLA (sBTLA) arises from the alternative splicing and cleavage of membrane-bound proteins. Initially recognized as a predictive biomarker in sepsis, sBTLA has gained attention for its potential to predict therapeutic efficacy in cancer patients. For instance, sBTLA has been reported to correlate with poor OS in non-responsive patients, while strong correlations have been observed among plasma levels of soluble IC proteins [223], including PD-1, PD-L1, and BTLA, in patients with PDAC. These findings suggest a shared origin and regulatory mechanism for these markers, with elevated levels linked to shorter OS. Establishing threshold values for each IC may differentiate patients with different OS, highlighting the potential of plasma IC levels in predicting survival and treatment efficacy in pancreatic cancer. Consistent with these findings, Wang et al. identified sBTLA as a potential prognostic factor for poor OS in patients with clear cell renal cell carcinoma (ccRCC) [224]. Furthermore, in HCC patients treated with sorafenib, elevated plasma sBTLA levels have emerged as an independent prognostic factor for advanced disease, correlating with reduced survival times. These findings highlight how plasma sBTLA levels influence the host’s antitumor immune response, ultimately affecting patient survival.

The balance of the immune response is further modulated by the HVEM/BTLA/LIGHT/CD160 axis, as described above. Interactions between HVEM on DCs or Tregs and either BTLA or CD160 trigger inhibitory signaling pathways, suppressing T-cell activation (Figure 4B) and proliferation by preventing the phosphorylation of TCR signaling molecules. These interactions dampen the antitumor immune response, allowing tumor cells to evade immune surveillance [225]. In contrast, HVEM engagement with LIGHT on T cells provides a stimulatory signal (Figure 4B), promoting T-cell proliferation, cytokine production, and strengthening of the antitumor immune response [105,226]. Therefore, the outcomes of HVEM interactions with BTLA or LIGHT within the TME are dichotomous: the BTLA-HVEM interaction promotes immune suppression and tumor progression, while the LIGHT-HVEM interaction drives immune activation and antitumor immunity. Thus, the regulation of HVEM, BTLA, LIGHT, and CD160 expression on immune cells plays a pivotal role in determining the degree of immune activation or inhibition, significantly influencing the immune response to cancer. Understanding the delicate balance of these interactions at the individual patient level is crucial for developing therapeutic strategies aimed at modulating the TME to enhance antitumor immune responses.

### 3.7. The VISTA/VSIG3/PSGL-1 Axis

The B7-CD28 superfamily member type I transmembrane protein Ig suppressor of T-cell activation (VISTA) is primarily expressed in hematopoietic cells [227]. Among leukocytes, myeloid cells exhibit the highest VISTA levels, particularly among microglia and neutrophils, with monocytes, macrophages, and DCs also showing significant expression [26]. In most cancers, among T cells, VISTA is especially prevalent in FOXP3^+^ CD4^+^ Tregs, contributing to promoting their differentiation and maintaining their phenotype while suppressing effector T-cell function (Figure 4B) [26,227,228].

VISTA comprises a single N-terminal Ig V-domain, along with a stalk of about 30 amino acids, a transmembrane region, and a cytoplasmic tail. The IgV domain resembles mostly PD-L1, while VISTA conserved cytoplasmic tail shares similarities with CD28 and CTLA-4, lacking the classic ITIM/ITAM motif typical of other B7 co-receptors. Instead, VISTA has a conserved SRC homology 2 (SH2)-binding motif, which may interact with STAT proteins, and three SH3-binding domains at the C-terminal end, two of which are found in CD28 and one in CTLA-4. While VISTA does not recognize ITIM or ITSM motifs, potential protein kinase C binding sites and a proline-rich motif that may serve as a platform for interactions with other complexes have been reported [26].

Recent studies have identified VISTA expression in the TME of human solid tumors, suggesting its potential as a prognostic biomarker for patient survival. However, the findings are mixed. Indeed, high VISTA levels have been related to improved OS in NSCLC, and a similar trend has been observed in esophageal adenocarcinoma, while in contrast, high VISTA expression has been linked to worse disease-specific survival (DSS), as reported by He et al. [229]. In their accurate systematic review and meta-analysis, He et al. have highlighted how, despite the controversial observations, high expression of VISTA in solid tumors is associated with favorable OS as compared with tumors with low expression levels [229], in association with higher CD8^+^ T-cell intra-tumor infiltration. However, as stated by the Authors, the limited number of studies examining the relationship between VISTA expression and patient prognosis in solid tumors makes it difficult to evaluate potential publication bias. To gain clearer insights, additional research is essential for a more comprehensive analysis.

Transcriptome analysis has revealed an involvement of VISTA in T-cell activation by influencing the TCR signaling and its downstream pathways, including the phosphorylation of PLC-γ, the dephosphorylation of LCK-pY505 by CD45, activation of ZAP-70, among others (Figure 4B) [230]. Moreover, VISTA expressed in APCs can influence their ability to present antigens to T cells. At variance, in a preclinical colorectal cancer model, VISTA blockade has been reported to impair the growth of small tumors, while in larger tumors resistant to anti-PD-1/CTLA-4 therapy, adding anti-VISTA led to tumor rejection in half of the cases, in association with increased antigen presentation, reduced myeloid-mediated suppression and increased interactions between T-cells and myeloid cells [231].

VISTA interacts with two key ligands, the V-set and Immunoglobulin Domain-Containing Protein 3 (VSIG3) (Figure 4B) [232], the glycoprotein ligand P-selectin 1 (PSGL-1) (Figure 4B) [233,234], and a less established partner, VSIG8 [235].

VSIG3, a member of the Ig superfamily, is expressed on various non-hematopoietic cells and has recently been recognized for its immunosuppressive effects. Although the exact mechanism of VSIG3 in suppressing tumor-associated macrophages (TAMs) and TILs remains unclear, it is expressed in several tumor types, such as gastric and hepatocyte cancers. While VSIG3 knockdown has been shown to inhibit tumor growth in vitro, its immunological role in tumors in vivo has yet to be confirmed. Structural and mutational analyses reveal that VSIG3 and PSGL-1 bind to overlapping but distinct regions on VISTA. Remarkably, VISTA binds to PSGL-1 exclusively at the acidic pH (Figure 4B) observed in the TME but not at the normal physiological pH [233], suggesting that PSGL-1 could be a binding partner for human VISTA in low-pH tumor settings. However, the role of PSGL-1 in antitumor immunity still needs to be fully understood, and direct evidence of its function is limited. While both VISTA and PSGL-1 can act as ligands and receptors, it is unclear whether their interaction occurs only in trans or cis to inhibit T-cell activation (Figure 4B). Notably, recent reports have highlighted an interaction between breast cancer and immune cells through the expression of the VISTA/VSIG3/PSGL-1 axis [236], suggesting its critical role in mediating the relationship between breast cancer cells and their microenvironment.

What emerges is a complex role for VISTA in tumor immunity, functioning both as an inhibitory and activating receptor yet impairing T-cell activation and inducing a quiescence state (Figure 4B) [231,234]. This proves essential to better investigate VISTA and its interacting partners as potential diagnostic and prognostic tools in cancer and clarify their roles in immune regulation. Notably, studies have shown that VISTA expression in immune cells can increase following IC inhibition in melanoma patients [237], suggesting that the negative regulation by VISTA may represent a critical potential mechanism of acquired resistance in cancer patients treated with PD-1 blockade.

The VISTA/VSIG3/PSGL-1 axis underscores the critical importance of analyzing the unique characteristics of a patient-specific TME. Abs targeting VISTA are undergoing clinical trials to treat various cancer types. CI-8993 has been assessed alone in a Phase I trial in patients with advanced solid tumor malignancies (NCT04475523) [238]; SNS-101 is being assessed in combination with cemiplimab (NCT05864144) [239], while HMBD-002 is currently being evaluated either as monotherapy or combined with pembrolizumab (NCT05082610) [240] (https://clinicaltrials.gov/, last accessed 2 November 2024). Combining these with acid-targeting drugs in the TME may also help counteract the immunosuppressive effects of the acidic environment and enhance the efficacy of ICIs and VISTA-targeting drugs.

## 4. Impact of Aging on the Cross-Talk Between Co-Stimulatory and Inhibitory Signaling in T Cells of Older Cancer Patients

In elderly cancer patients, the TME and the immune system generally experience significant alterations due to aging. These changes include chronic inflammation (inflammageing) [241] and immunosenescence, a change in terms of phenotype and function of immune cell populations leading to the onset of the senescence-associated secretory phenotype (SASP), characterized by high levels of cytokines and chemokines and including IL-6, IL-8, IL-15, CXCL1, CCL3, and others [242]. This SASP phenotype can either support antitumor responses or encourage an immunosuppressive cell infiltration, including myeloid-derived suppressor cells (MDSCs), Tregs, M2 macrophages, and N2 neutrophils, thus contributing to the generation of an immunosuppressive milieu.

In the context of TME in elderly patients, the balance between co-stimulatory and inhibitory molecules may become disrupted, which can affect antitumor responses. Older individuals often exhibit unique patterns of PD-L1 expression, varying degrees of TIL infiltration, and heightened T-cell exhaustion, marked by the upregulation of PD-1, TIM-3, PD-1, and LAG-3 Recent research has also reported that CTLA-4 expression tends to increase with age, potentially contributing to immune dysregulation in the elderly [243]. These alterations in TIL functionality associated with aging can potentially impact the efficacy of ICIs and influence the overall immunotherapy response and biomarkers in older patients. These immune regulatory changes could significantly alter how older patients respond to immunotherapy treatments.

At the same time, co-stimulatory signals may be impaired. In particular, CD28 is universally expressed on T cells from younger subjects, while declining with age [58,244], along with the expression of CD27, leading to a “senescent” phenotype, portrayed by terminally differentiated T cells characterized by shortened telomeres [58].

The evidence regarding ICOS expression in relation to aging is less consistent. Some studies have shown a decrease in ICOS expression in T cells from older individuals compared to their younger counterparts. At variance, other research suggests that ICOS may represent a compensatory mechanism for the loss of CD28 during progressive population doublings, ultimately emerging as the primary co-signaling molecule in this scenario [245].

The effects of aging on OX40 signaling in T cells are also intricate. In particular, while in an ovalbumin (OVA)-expressing tumor model, the treatment with an OX40 agonist reduced the differentiation of T cells into effectors aged mice compared to younger mice [246], other studies have revealed that T cells transferred into aged mice exhibit heightened OX40 signaling and activation [247]. Notably, as highlighted by these Authors, Tregs express OX40 at significantly higher levels than non-Treg CD4^+^ T cells. Therefore, systemic OX40 stimulation in aged mice might elevate Treg populations, which can suppress the differentiation of effector T cells.

Remarkably, IL-6, heavily produced during inflammation, has been identified as a marker associated with poor responses to atezolizumab (an anti-PD-L1 therapy) in large-scale clinical trials involving advanced kidney, breast, and bladder cancers. In preclinical studies, the simultaneous blockade of PD-L1 and the IL-6 receptor (IL6R) leads to a synergistic reduction in large tumors and significantly enhances CD8^+^ CTL responses compared to treatment with an-ti-PD-L1 alone [248].

These changes can create an environment where inhibitory signals dominate over co-stimulatory signals, potentially leading to T-cell exhaustion or dysfunction and thereby impairing both effective antitumor responses and responses to ICIs [249]. This consideration is particularly important when administering ICB in older patients.

## 5. Concluding Remarks: Achieving Optimal Immune Responses Through Favoring Fine-Tuned Responses

Combining ICIs with co-stimulatory molecule agonists represents a promising strategy for enhancing antitumor immunity by simultaneously blocking inhibitory pathways and promoting robust T-cell activation. However, the efficacy of these combinations is intricately linked to the complex and dynamic interplay between co-stimulatory and inhibitory molecules within the TME.

Initial trials combining co-stimulatory agonists and ICIs have demonstrated safety but have yet to produce the expected strong antitumor effects. This suggests that, while the approach holds promise, the complexity of the TME requires more refined and tailored studies that consider specific immune interactions and the deriving T-cell responses. Thus, a comprehensive analysis of the expression patterns of key co-stimulatory and inhibitory molecules and their roles and interactions in the TME is essential for fully understanding the functional consequences of these molecules at a single patient level. A personalized approach that takes into account the heterogeneity of the TME, both between patients and within different regions of the same tumor, is crucial and will help ensure that treatments are tailored to the unique immunological landscape of each tumor. This could maximize therapeutic efficacy while minimizing unnecessary toxicity.

Moreover, a deeper understanding of the molecular interactions within these pathways may provide critical insights for identifying robust biomarkers, which can guide treatment decisions.

As cancer immunotherapy continues to evolve, the integration of these molecular insights into clinical practice will be critical to improving the outcomes of personalized medicine. Future research should focus on improving evaluative techniques. Recent advancements in spatial profiling technologies have provided powerful new tools for investigating the physical organization of cells within the TME, mainly through techniques like single-cell RNA sequencing (scRNA-seq) [250]. These technologies can provide critical insights by mapping the spatial distribution of co-stimulatory and inhibitory molecules across heterogeneous tumor regions, often characterized by distinctive cellular compositions.

Moreover, tools like CellChat, a database specifically designed to quantitatively analyze intercellular communications from scRNA-seq data [251], can potentially explore complex ligand-receptor interactions within the TME, facilitating the examination of the dynamics of T-cell cross-talk. These advances will enable the development of more tailored immunotherapies, finely tuned on the unique tumor and immune landscape of each patient.

Exosomes and microvesicles, which carry inhibitory or co-stimulatory molecules, also play a critical role in modulating the response to ICIs. Tumor-derived exosomes (TDEs) are particularly influential, as they can either directly deliver PD-L1 [252] or enhance its expression in stromal and immune cells such as TAMs [253]. This upregulation of PD-L1 promotes immune evasion by reducing T-cell activity, weakening the effectiveness of ICIs targeting the PD-1/PD-L1 axis and creating an immune-resistant TME. Furthermore, T-cell exhaustion, induced by TDEs, limits T-cell responsiveness to ICIs, underscoring the need to address the mechanisms driving this exhaustion.

In addition, exosomal microRNAs, like the miRNA-424 derived from colon cancer cells, have been reported to suppress the critical CD28-CD80/86 co-stimulatory signals necessary for T-cell activation [253]. This disruption of co-stimulatory signaling compromises the therapeutic potential of ICIs, which rely on intact co-stimulatory interactions to restore antitumor immunity. Tumor-derived EVs can also carry FASL, which induces apoptosis in T cells and NK cells via the FAS-FASL pathway, further diminishing antitumor responses. Moreover, EVs carry TGF-β, a potent immunosuppressive molecule that activates Tregs and MDSCs, contributing to immune suppression [252]. In addition, EVs induce an M2-like immunosuppressive macrophage phenotype through miR-934 derived from CRC, which promotes liver metastasis [253]. Other factors carried by EVs, such as arginase 1, GAL-9, and adenosine (produced via CD39 and CD73), amplify these immunosuppressive effects within the TME [252]. By delivering PD-L1 and other inhibitory molecules, EVs may directly compete with ICIs by sequestering therapeutic Abs or engaging immune cells, thereby impairing the efficacy of ICB therapies.

Finally, to fully understand the mechanisms underlying the therapeutic outcome following ICIs, it is essential to consider the impact of IC expression in cellular compartments beyond T cells within the TME. As reported above, a significant concern is the expression of various co-stimulatory and inhibitory molecules across distinct cell subsets within the TME [3,23]. While anti-PD-1 therapies primarily focus on enhancing T-cell activity to combat tumors, emerging evidence suggests that PD-1 expression on tumor cells themselves plays a critical role in cancer progression. Notably, Rotolo and colleagues have demonstrated that in NSCLC, PD-1 expression is observed on tumor cells, particularly following cisplatin treatment, where it correlates with chemoresistance and stem-like features [254]. These PD-1-positive tumor cells exhibit resistance to chemotherapy and are responsive to anti-PD-1 Abs even in the absence of lymphocyte involvement. This highlights the potential of tumor cell-intrinsic PD-1 expression as a novel predictive biomarker for the efficacy of anti-PD-1 therapy, offering a new therapeutic avenue for treating NSCLC [255].

Conversely, PD-1 blockade has also been associated with the proliferation of highly suppressive PD-1^+^ effector Tregs in cases of hyperprogressive disease (HPD), thereby inhibiting antitumor immunity [256]. Actively proliferating PD-1^+^ effector Tregs in tumors have been identified as reliable markers of HPD, underscoring the complexity of PD-1-targeted therapies. Additionally, mediators in the TME, such as lipopolysaccharides (LPS), can induce PD-1 expression on MDSCs [257], which are potent suppressors of antitumoral immune responses. By targeting PD-1 expression on these cells, ICIs can remodel the myeloid compartment, shifting it from an immunosuppressive to a pro-inflammatory state and thereby enhancing antitumor T-cell responses.

Moreover, CTLA-4 expression, while predominantly described in T cells, has also been observed in a variety of cell types, including tumor cells, DCs, and other cells of lymphoid and myeloid lineages [258]. Unlike the membrane-associated CTLA-4 on T cells, CTLA-4 expression in DCs is diffusely distributed, with significant localization to the trans-Golgi network. CTLA-4 in DCs is subsequently packaged into EVs and exported. These EVs can be taken up by bystander DCs in a CD80/CD86-dependent manner, leading to the downregulation of surface B7 molecules and diminishing the ability of APCs to activate T cells [258].

These findings underscore the complexity of IC dynamics within the TME and suggest that successful immunotherapy strategies must also account for their diverse roles across various cell types. A comprehensive understanding of these mechanisms will be essential for optimizing current therapies and developing innovative approaches to overcome resistance and improve patient outcomes.

## Data Availability

Not applicable.
